# Photodissociation Dynamics of Formic Acid at 230 nm: A Computational Study of the CO and CO_2_ Forming Channels

**DOI:** 10.1021/acs.jpca.4c07933

**Published:** 2025-01-16

**Authors:** Yi-Sin Ku, Po-Yu Tsai

**Affiliations:** Department of Chemistry, National Chung Hsing University, Taichung 402, Taiwan

## Abstract

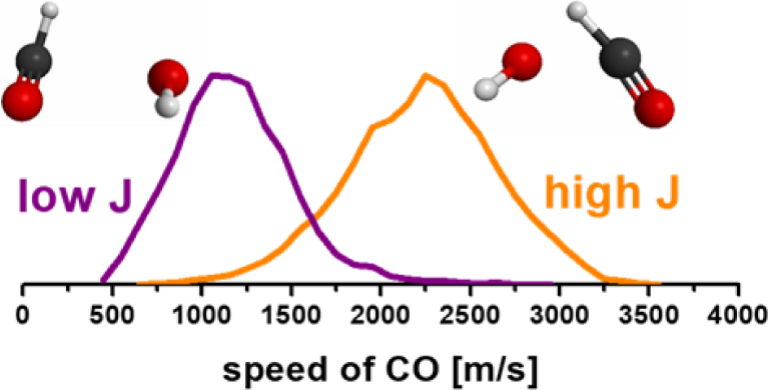

Recent photolysis experiments with formic acid suggest that the roaming mechanism is a significant CO-forming pathway at a photolysis energy of 230 nm. While previous computational studies have identified multiple dissociation pathways for CO-forming channels, the dynamic features of these pathways remain poorly understood. This study investigates the dissociation dynamics of the CO + H_2_O and CO_2_ + H_2_ channels in the ground state (S_0_) of formic acid using direct dynamics simulation and the generalized multi-center impulsive model (GMCIM) at 230 nm. Computational results summarize the characteristics of the product states from six different dissociation pathways, including two roaming pathways. A comparison of the simulated speed distribution of CO products with experimental observations shows that high-rotational CO products predominantly originate from the three-center dissociation pathway. Furthermore, while experimental results reveal a bimodal speed distribution of CO at low rotational states, our findings suggest that the OH roaming pathway contributes to the fast component of this distribution, rather than the slow component. Furthermore, another isomerization-mediated four-center pathway contributes negligibly to the experimental results. The agreement between computational results and experimental observations at 230 nm supports the previously proposed dissociation mechanism of the CO + H_2_O channel. For the CO_2_ + H_2_ channel, this study provides useful information for experimental identification of dissociation pathways in the future.

## 1 Introduction

The role of the roaming mechanism in unimolecular dissociation has been extensively investigated over the past two decades. The roaming mechanism has been observed in photolysis experiments of many compounds, such as aldehydes,^1−7^ acetone,^8,9^ alkanes,^10^ nitrate radical,^11^ nitronitrites,^12^ methylmethane,^13^ methyl formate,^14,15^ and formic acid.^16–18^ The importance of roaming mechanisms in other molecules has also been investigated, for example, MgH_2_,^19^ nitrobenzene,^20,21^ methanol,^22^ and methylamine.^23,24^ Several papers discuss the importance and physical interpretation of the roaming mechanism.^25−34^ The appearance of the roaming mechanism prompts researchers to focus on overlooked regions of the multidimensional potential energy surface (PES). With the existence of the roaming mechanism, it has been shown that the dissociation mechanism consists of multiple competitive dissociation pathways. According to recent studies, the roaming mechanism can occur not only on a single adiabatic PES but also on multiple electronic states with the aid of nonradiative transitions between multiple adiabatic PESs.^11,14,17,18,35,36^ In some cases, the conical intersection on the PES is believed to play a key role in facilitating the roaming mechanism.^14,18^ In most cases, the discovery of the roaming mechanism in a compound is initially driven by experimental observations, which are later supported by computational studies. Although the concept of the minimum energy path (MEP) is not essential to characterize the roaming mechanism,^30,37,38^ there are instances where the MEP of the roaming mechanism is predicted by calculations before experimental verification. The roaming dissociation mechanism of formic acid is one such case.^16−18^

Formic acid, the simplest carboxylic acid, is known for its roles in chemical synthesis, combustion, and atmosphere chemistry. Previous experimental studies on the photolysis of formic acid have primarily focused on wavelengths between 220 and 250 nm.^39−52^ Within this range, the absorption cross-section of formic acid increases at shorter wavelengths, while its fluorescence decreases due to the opening of several radical dissociation pathways.^17,44,51^ The dissociation mechanism and energy thresholds of free radical channels have been determined experimentally, including the breaking of O–H and C–H bonds, as well as the formation of OH radicals.^39−41,43−45,51^ With the aid of experimental observations, previous studies suggest that these radical products are primarily generated through dissociation pathways on the S_1_ and T_1_ PES.^17,45,47,51,53^ Furthermore, two molecular dissociation channels, CO + H_2_O and CO_2_ + H_2_, were observed in experiments,^16,47,48,54^ both of which correlate with the S_0_ PES of formic acid. In relevant studies, it was observed that the branching ratio of CO and CO_2_ depends on the cis–trans isomerism of formic acid, a phenomenon referred to as conformational memory.^17,48,54−56^ Theoretical calculations have identified possible dissociation pathways for both the radical and the molecular channels.^17,18,47,53,55−59^ Multiple dissociation pathways exist on the S_0_ PES for both CO and CO_2_-forming channels,^17,18^ but earlier computational studies identified only the pathway with the lowest energy threshold for each molecular channel.^47,53,55−59^ The saddle points are three-center and four-center types for CO- and CO_2_-forming channels, respectively.^17,57−59^ On-the-fly direct dynamic simulations were performed to investigate the product state distributions of these two pathways at photolysis wavelengths of 248 and 193 nm.^57^ More than a decade ago, Maeda et al. conducted a systematic theoretical study to explore the adiabatic PESs of the S_1_, T_1_, and S_0_ states using global reaction path searching methods.^17,18^ They identified all possible dissociation pathways for the molecular and radical channels, located the minimum energy conical intersection (MECI) structures between singlet PESs, and the minima on the seam of crossing (MSX) structures between singlet/triplet PESs. In addition to explaining the effect of conformational memory via photochemical pathways on multistate PESs, they discovered several new dissociation pathways for the CO and CO_2_-forming channels.^17,18^ Among these new pathways, a few relate to the roaming mechanism of CO and CO_2_-forming channels, which had not been observed experimentally at that time. In the recent state-specific ion imaging experiments at 230 nm conducted by Wang’s group,^16^ the speed distributions of CO products were measured at different rotational states. They found that the speed distributions of CO at high rotational states differ from those at low rotational states. For low rotational CO, the products exhibit bimodal speed distributions, with both components being slower than those of high rotational CO.^16^ The result of low-speed, low-rotation CO products serve as evidence of the roaming mechanism in the CO-forming channel of various molecules.^1,2,8,15^

This study was motivated by the following reasons:

(1) Although direct dynamic simulations were performed for the CO + H_2_O and CO_2_ + H_2_ channels at 248 and 193 nm in 2005,^57^ several newly discovered dissociation pathways for these two channels were reported later in 2012.^17,18^ The dynamical characteristics of these new pathways remain unknown. While the most probable dissociation mechanism can be deduced from static data of saddle points and minimum energy paths (MEPs), the dynamic characteristics of the products from all possible pathways are necessary to evaluate the proposed mechanism with experimental evidence.

(2) The roaming mechanism of the CO + H_2_O channel was examined experimentally at 230 nm in a recent study by Wang et al.^16^ but the corresponding dynamical calculation at 230 nm has yet to be performed.

Given that several new dissociation pathways of molecular channels have been discovered,^17^ further dynamical calculations incorporating these pathways are necessary to interpret recent experimental results.^16^ The experimentally observed CO products^16^ exhibit a bimodal speed distribution at low rotational states, with both components being slower than those observed at high rotational states. Although slow-moving products may suggest the existence of a roaming mechanism, it remains unknown how slow a roaming product should be. Consequently, dynamical calculations are still required to determine which component is contributed by roaming mechanism at low rotational states.

In this study, we employed on-the-fly direct dynamic simulations of classical trajectories for six different pathways of the CO + H_2_O and CO_2_ + H_2_ channels at 230 nm. Four of these pathways have never been computationally investigated before. The simulations were performed on the S_0_ adiabatic PES at the computational level of density functional theory (DFT). Additionally, we utilized the generalized multicenter impulsive model (GMCIM),^60−62^ a previously developed kinematic model, to predict the characteristics of product states for each dissociation pathway. Based on the assumption of a sudden limit, the GMCIM decomposes product energies into two contributions: the potential energy gradient along the dissociation coordinate and the transverse mode vibrations, aiding in the explanation of the dynamic features of different dissociation pathways.^60,61^ All of these dynamical calculations were initiated from the barrier top of each pathway, without considering the dynamics before the transition states or the competition between different pathways.

The remainder of this paper is structured as follows: Section 2 describes the electronic structure calculations and on-the-fly direct dynamics simulations and briefly reviews the basic concept of the GMCIM. Section 3 presents the product state distributions from the simulations and compares them with previous studies. Section 4 discusses the characteristics of dissociation pathways and the dissociation mechanism. Finally, Section 5 provides the conclusion of this paper.

## 2 Methods

### 2.1 Electronic Structure Calculation

The optimization of the saddle point (SP) structures, harmonic frequency analyses, and intrinsic reaction coordinates (IRC) calculations were performed using the GAMESS 2020R2 package.^63,64^ A total of six different saddle points and their corresponding dissociation pathways on the S_0_ surface of formic acid were investigated in this study, including two loose saddle points associated with roaming-type dissociation pathways.^17^ In previous computational studies of the S_0_ state surface of formic acid,^17,47,53,57−59^ saddle point structures for various CO + H_2_O and co + H_2_ dissociation pathways were identified using single-reference and multireference electronic structure methods. For direct dynamics simulations, we reproduced these dissociation pathways using DFT in this study. The geometries of all the saddle points were optimized at the M06-2X/aug-pcseg-1 level. The effects of basis set size on the saddle point geometries and exit barrier heights were carefully evaluated. The electronic structures of roaming saddle points exhibit diradical character, thus, multireference methods are typically required,^25,26,65^ to locate these saddle points. By employing spin-unrestricted density functionals that include a significant contribution of exact Hartree–Fock (HF) exchange at long-range, it is possible to locate the roaming saddle point with acceptable geometry and barrier height.^61,66^ Therefore, we investigated the roaming saddle points using spin-unrestricted M06-2X density functionals, with the HOMO and LUMO mixed in the initial guess. Vibrational frequency analyses were conducted by computing the numerical Hessian. IRC calculations were performed using the Gonzalez–Schlegel second-order method (GS2)^67^ with a step size of 0.05 amu^1/2^-Bohr.

### 2.2 Direct Dynamic Simulation

Direct dynamics simulations were conducted for six different dissociation pathways. Classical trajectories were integrated on-the fly with potential energies and gradients obtained directly from electronic structure calculations at the M06-2X/aug-pcseg-1 level. The simulations were performed using a modified version of the VENUS/NWChem software package.^68^ The modification was necessary to ensure the HOMO–LUMO mixing of the initial guess at the beginning of each trajectory, generating spin-unrestricted singlet electronic states for the roaming pathways on the S_0_ potential energy surface of formic acid. The results reported in this study are based on approximately 32 000 classical trajectories obtained through direct dynamics simulations from various dissociation pathways at three different total energies. Except for the two roaming pathways (TS4 and TS5), where trajectories were initiated a few steps beyond the loose saddle points in the plateau region of the IRCs, classical trajectories for nonroaming pathways were initiated at one of the saddle points. Trajectory simulations were carried out with time steps of 0.5 fs. The maximum duration of a single trajectory was 400 fs for roaming pathways and 300 fs for other pathways. The variation in total energy was approximately 1 kcal/mol during the integration of the trajectories. At each time step, the classical equations of motion were integrated numerically using the velocity–Verlet algorithm. The dissociation criterion was defined as a relative distance of 6 Å between the pairs of molecular products (CO + H_2_O or CO_2_ + H_2_). No significant changes in the product state energies were observed when increasing the relative distance used as the criterion for successful dissociation.

In the dynamic simulations, the initial energy above the barrier top for each pathway was calculated as the sum of the photon energy and the harmonic zero-point energy (ZPE) at the global minimum of trans-form formic acid (denoted as MIN1 in reference (17)), minus the barrier height from MIN1 to the saddle point. Direct dynamics simulations were performed at three different total energies: (1) ZPE + barrier height of each pathway, (2) ZPE + 230 nm photon energy, and (3) ZPE + 248 nm photon energy. For a given total energy, the initial energy above the barrier top of a dissociation pathway was obtained by subtracting its barrier height from the total energy. Initial conditions were generated by microcanonical quasi-classical sampling at the starting geometry.^69,70^ At a given initial energy, the energy of each vibrational mode was sampled according to its density of states. The initial displaced coordinates and momenta were determined by randomly sampling the initial phase of each normal mode. After sampling all the 3N–7 vibrational modes, the residual initial energy was assigned to the dissociation coordinate, expressed as velocity components directed toward the products. The rotational angular momentum of the parent molecule was assumed to be zero.

### 2.3 The Generalized Multi-Center Impulsive Model

We briefly review the GMCIM; detailed descriptions of this model have been reported in previous studies.^60−62^ For each dissociation pathway, GMCIM focuses on the shape of the multidimensional PES around the MEP. In this framework, molecular dissociation on a multidimensional PES can be reduced to an effective one-dimensional problem along the dissociation coordinate, with the remaining 3N–7 transverse vibrational modes orthogonal to it. Then the sudden approximation is introduced to the dissociation coordinate by assuming that atomic motions along the transverse modes are slow and, therefore, remain frozen during the dissociation.

The GMCIM begins at the saddle point of a dissociation pathway. A set of initial atomic displacements and velocities is provided to initiate dissociation, representing the potential and kinetic energy components of the initial energy, respectively. Similar to actual classical trajectory calculations, the initial conditions are generated by microcanonical quasi-classical sampling of the 3N–7 transverse modes at a given initial energy. The model then considers the effect of the exit barrier along the dissociation path on the initial velocities by calculating the acceleration vector due to the gradient of the exit barrier.^60^ Since the dissociation path may not be linear on the PES, the dynamics along the curved dissociation coordinate are treated as successive events of constant-acceleration motion along an approximate zigzag path, achieved by linearly interpolating the IRC points.^60^ The acceleration vector is calculated from the mass-weighted Cartesian gradients at each IRC point. The changes in product states due to the gradient are calculated and accumulated at each IRC step until the contributions from the entire IRC are accounted for. The potential energy contributions of the 3N–7 transverse modes are treated using the sudden approximation.^61^ Unlike their kinetic energy counterparts, which interact with the gradient vectors along the dissociation coordinate, the potential energy contribution is incorporated into the product states separately. GMCIM employs a purely impulsive approach rather than assuming the commonly used statistical/impulsive hybrid scheme.^61^ The model divides the transverse modes into two categories: (i) conserved modes, which correlate to product vibrations at the end of the dissociation and (ii) transitional modes, which correlate to the translation of products and disappear at the end of dissociation. Conserved and transitional modes can be identified by examining the mass-weighted Cartesian eigenvector of each transverse mode.^61^ The formulas for gradient accumulation, product state analysis, and the treatment of the 3N–7 transverse modes have been described in detail in previous studies.^60,61^

In practice, a large number of initial conditions are required to form smooth product state distributions. At least 2,000 different initial conditions were generated for each dissociation pathway at a specific photolysis wavelength. The data required for GMCIM include the geometry and gradient at each IRC point, as well as the nuclear Hessian at the saddle point. These data can be obtained by performing typical electronic structure calculations, such as vibrational frequency analysis and IRC calculations for a saddle point, using the same methods and basis sets applied in direct dynamics simulations. The formulas for GMCIM are suitable for efficient array programming and are currently implemented in a program written in MATLAB.^71^ GMCIM differs from actual classical trajectory simulations. Molecules are assumed to remain within the local region of the PES near the minimum energy path, and the model does not account for the possibility of molecules rebounding or revisiting previous IRC points. Additionally, GMCIM does not consider energy flow between the dissociating product pairs after the energy released from the exit barrier is obtained. In other words, GMCIM does not account for final state interactions but ensures that the outcomes strictly adhere to kinematic constraints.

## 3 Results

### 3.1 Electronic Structure Calculation

Previous computational studies^17,18,47,53,55−59^ have thoroughly explored the S_0_ state PES of formic acid. To verify the reliability of our adopted methods and basis sets, we repeated these computations, achieving a balance between computational efficiency and accuracy for use in on-the-fly direct dynamics simulation. The spin-unrestricted M06-2X density functional was used in all electronic structure calculations, due to its strong performance in predicting barrier heights and thermochemistry of chemical reactions,^72^ as well as its ability to include roaming saddle points in the PES.^61,66^

A previous theoretical study^17^ identified eight different dissociation pathways correlating to either the CO + H_2_O or CO_2_ + H_2_ channels on the S_0_ PES. The notation from reference (17) is adopted in this study to label these saddle points. Four of the eight pathways correlate to the CO + H_2_O channel: TS2, TS14, TS5, and TS15. The remaining four pathways, TS9, TS16, TS4, and TS6, correspond to the CO_2_ + H_2_ channel. Four of these saddle points^17^ exhibit characteristics of the roaming mechanism, indicated by extensive elongation of one bond in their geometries: TS5 (HCO--–OH), TS15 (H---COOH), TS4 (H---COOH), and TS6 (HCOO---H). Among these pathways, saddle points TS15 and TS6 could not be successfully located using DFT calculations; therefore, the results of the direct dynamics simulation are only presented for the remaining six pathways. Nevertheless, a brief description of TS15 and TS6 will be provided later.

**Figure 1:**
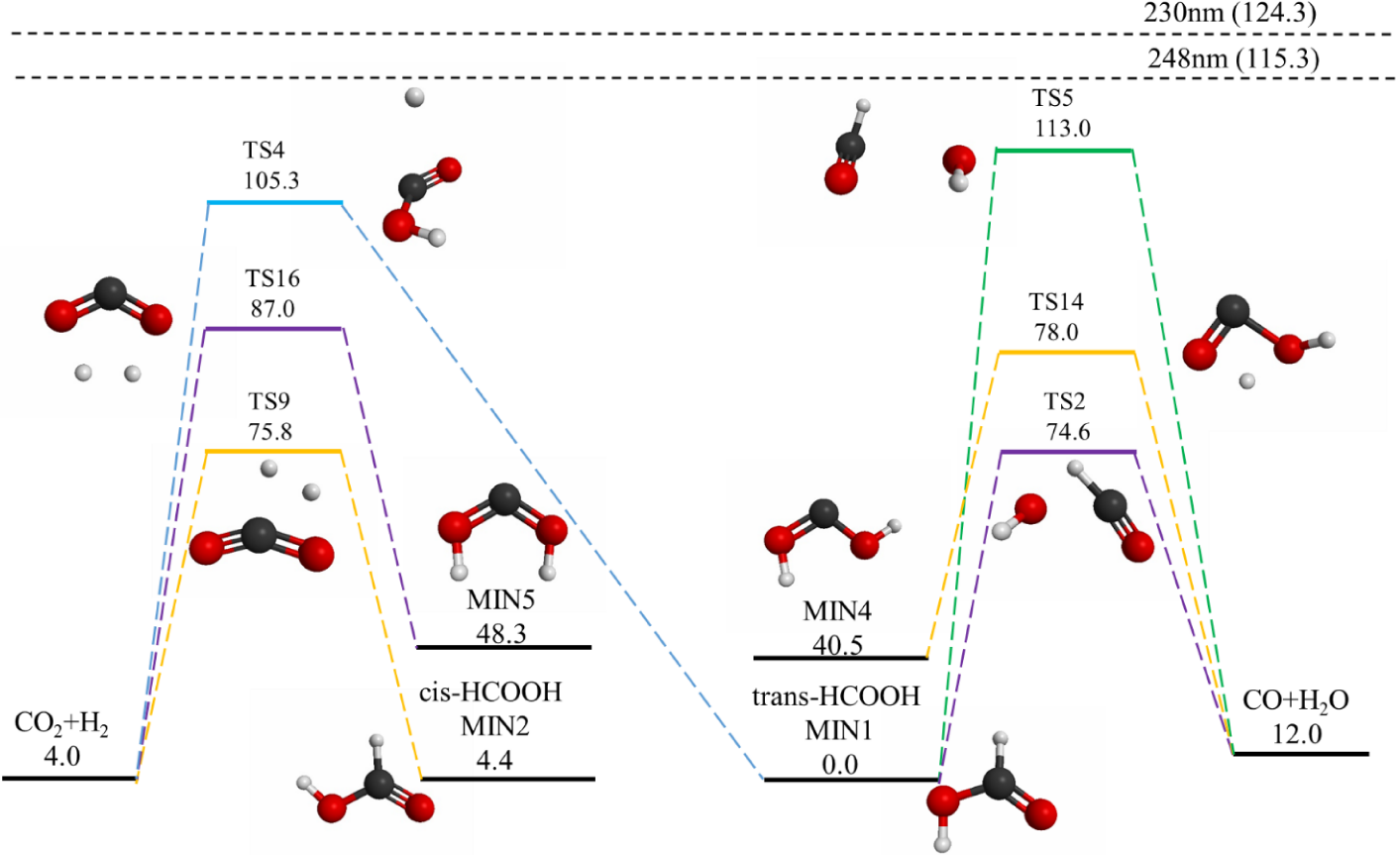
Energy level diagram (unit in kcal/mol) of the six dissociation pathways calculated at the M06-2X/aug-pcseg-1 level.The energy values of each saddle point and product relative to the trans-form of formic acid are reported without considering the zero-point energy.

The energies of the stationary points related to the CO and CO_2_ forming pathways on the S_0_ state PES of formic acid, calculated using the M06-2X functional with various basis sets, are presented in Figure 1, Tables 1, and S1 (Supporting Information). The energies of these stationary points are reported relative v to the trans-form minimum of formic acid (MIN1^17^), with all stationary points labeled according to the notation adopted from reference (17). Jensen’s segmented contracted polarization consistent basis sets (pcseg-n or aug-pcseg-n)^73^ were used in this study. Theaug-pcseg-1 basis set employed for calculating the relative barrier heights produced results comparable to those obtained using the pcseg-2 and aug-pcseg-2 basis sets, with an average deviation of less than 1 kcal/mol in average (Table S1). The effect of the basis sets on the geometric parameters was also minimal. As shown in Table S2, with geometric parameters defined in Figure S1, the bond length variation across different basis sets was less than 0.02 Å for TS2 and TS5. The bond angles exhibited minimal variations as well, except for the dihedral angles of TS5, which showed a variation of 10° among different basis sets. Based on these findings, the aug-pcseg-1 basis set provided a reasonable compromise between computational efficiency and accuracy for performing-direct dynamics simulations. When compared with previous studies, the results of our M06-2X calculations demonstrated qualitative agreement with more sophisticated calculations using MS-CASPT2/aug-cc-pVDZ^17^ (Table 1). The M06-2X functional reproduced the general trend in the relative energy differences between saddle points observed in earlier CASPT2 calculations: TS2 < TS9 < TS14 < TS16 < TS4 < TS5. Some of the saddle points show good agreement in their barrier heights (e.g., TS9 and TS14), while others exhibit differences of 4–6 kcal/mol (e.g., TS2, TS16, TS5, and TS4). In general, the results of the M06-2X functional predict higher barrier heights than those obtained with CASPT2. The mean difference between the barrier heights calculated using M06-2X/aug-cc-pcseg-1 and CASPT2/aug-cc-pVDZ^17^ was approximately +4 kcal/mol. According to the data in Tables S2 and S3, with geometric parameters defined in Figure S1, the differences in geometric parameters between the two methods were minimal. The mean difference in bond lengths was less than 0.03 Å, and the mean difference in bond angles was less than 2°. The geometric parameters of the CASPT2 calculations were obtained from the Cartesian coordinates provided in the Supporting Information of ref (17)).

**Table 1:** Energies in kcal/mol, of the Stationary Points Relative to trans-HCOOH (MIN1) Calculated by Using M06-2X/aug-pcseg-1 and CASPT2/aug-cc-pVDZ

	**MIN1**	**MIN2**	**MIN4**	**MIN5**	**CO + H**_**2**_**O**	**CO**_**2**_**+ H**_**2**_
**M06-2X**	0	4.4	40.5	48.3	12.0	4.0
a**CASPT2**	0	4.2	43.4	51.2	7.8	4.2
	**TS2**	**TS4**	**TS5**	**TS9**	**TS14**	**TS16**
**M06-2X**	74.6	105.0	113.0	75.8	78.0	87.0
a**CASPT2**	70.7	98.9	107.2	73.7	77.6	81.0

a Results adopted from ref (17).

### 3.2 Dissociation Pathways

**Figure 2:**
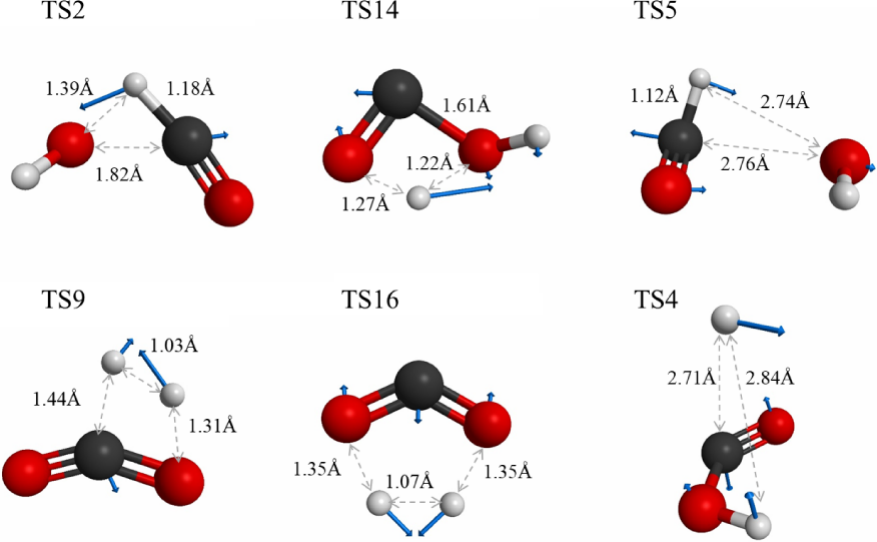
Structure of six saddle points calculated at the M06-2X/aug-pcseg-1 level. The Cartesian vector of the imaginary modes of saddle points are also shown.

The imaginary mode of each saddle point provides insight into the dynamic features of the corresponding dissociation pathway, even though the gradient vector of the exit barrier along the dissociation coordinate may not align perfectly with the imaginary mode. The saddle-point structures examined in this study, including the normal-mode vector of the imaginary vibrational mode, are illustrated in Figure 2.

#### 3.2.1 CO + H_2_O Pathways

##### 3.2.1.1 TS2

TS2 is a typical three-center saddle point, similar to those found in aldehydes, acetones, and esters.^9,61,65^ TS2 connects the trans-form parent molecule (MIN1, Figure 1) to the CO + H_2_O product. During the transition to the molecular product, the parent molecule undergoes concerted breaking of the C–OH and C–H bonds while forming a new HO–H bond. Consequently, the impulse acts solely on the C, O, and H atoms. This type of impulse accelerates the two-body molecular products during their recoil, resulting in a high-speed CO + H_2_O product.^57^ Additionally, since the impulse is exerted exclusively on the carbon atom of the CO product, the CO molecule exhibits high rotational excitation. Meanwhile, the H_2_O products receive moderate vibrational excitation due to the newly formed OH bond.

##### 3.2.1.2 TS14

TS14 is a four-center saddle point that connects an isomer of the parent molecule (MIN4, Figure 1) to the CO + H_2_O product. To form the molecular product via this pathway, the parent molecule must undergo isomerization before dissociation. The four-center impulse arises from the concerted breaking of the CO–H and OC–OH bonds and the formation of the H–OH bond. Because both atoms of the CO moiety are involved in the bond rupture, the torque exerted on the CO moiety is smaller than that of TS2. Therefore, one can expect high-speed products with low rotational excitation for the CO molecule.

##### 3.2.1.3 TS5

TS5 is a loose saddle point of the HCO–OH roaming pathway that connects MIN1 to the CO + H_2_O product. To reach this saddle point from MIN1, the C–OH bond length increases to over 2.5 Å, at which point the HCO moiety rotates along its CO axis, transferring the H atom to the OH moiety. The impulse generated during the formation of the H–OH bond leads to a high vibrational excitation of the H_2_O product. Consequently, the products exhibit low translational speeds, and the CO molecule receives limited internal excitation. Theoretically, the roaming mechanism can be demonstrated on a PES even in the absence of a roaming saddle point.^26,30,33,34,37,38,65^ This is because the roaming dynamics are not solely determined by the characteristics of the PES.^26,33,38^ Locating the roaming saddle points, allows us to identify pathways toward the exit barrier, which highlights the dynamical characteristics of the roaming mechanism.

##### 3.2.1.4 TS15

Similar to TS5, TS15 is also a roaming saddle point that leads to the CO + H_2_O product,^17^ differing only in the roaming schemes (HOCO–H) that correlate with MIN4 (Figure 1). This saddle point could not be located using single-reference DFT calculations, leaving its detailed dynamic signatures unknown. A previous study reported that TS15 is approximately 10 kcal/mol lower in energy than TS5.^17^

#### 3.2.2 CO_2_ + H_2_ Pathways

##### 3.2.2.1 TS9

TS9 connects the cis form of formic acid (MIN2, Figure 1) to the H_2_ + CO_2_ product. The four-center impulse originates from the concerted breaking and formation of the C–H, O–H, and H–H bonds. Consequently, one would expect to observe high-speed products with low-rotational excitation and moderate vibrational excitation of H_2_. For the CO_2_ + H_2_ pathway, the geometry of the CO_2_ moiety must change from bent (at the saddle points) to linear (CO_2_ product). Consequently, part of the energy released from the exit barrier contributes to this geometric transformation, leading to non-negligible vibrational excitation in the CO_2_ products, regardless of the dissociation pathway.

##### 3.2.2.2 TS16

TS16 connects isomer MIN5 (Figure 1) to the H_2_ + CO_2_ product. Due to its symmetric structure and four-center impulse, the rotational excitation of the product pair is negligible. High-speed, vibrationally excited products are expected from this pathway.

##### 3.2.2.3 TS4

TS4 is a loose saddle point of the HOCO–H roaming pathway, which resembles the geometry of the HCOO + H radical dissociation and connects MIN1 to the CO_2_ + H_2_ product. Similar to the roaming pathway of TS5, the C–H bond length of trans-form formic acid increases until reaching a plateau region on the PES, where the bond distance exceeds 2.5 Å. Simultaneously, the COOH moiety must reorient to arrive at TS4. The roaming hydrogen then approaches the H-end of the OH moiety for the final abstraction of the H_2_ product. Due to the characteristics of the MEP of this pathway (Figure S2), the impulse exerted by the exit barrier is equally divided between the formation of H–H bonds and the departure of the dissociating products. This behavior differs from the typical roaming dissociation mechanism,^25,26,34^ such as that observed in TS5. Consequently, one might expect not only significant vibrational excitation of H_2_ but also moderate energy excitation in other product states.

##### 3.2.2.4 TS6

Similar to TS4, TS6 is also a roaming saddle point that leads to the CO_2_ + H_2_ product, but with a different roaming hydrogen atom, resembling the geometry of the H + COOH radical dissociation. Both roaming saddle points correlate to trans-form formic acid (MIN1), although the TS6 saddle point geometry features an elongated O–H bond and is therefore more similar to several S_0_/S_1_ MECI structures.^17,18^ Thus, it is believed that the TS6 pathway may serve as a destination for the “frustrated radical dissociation” of HCOO + H on the S_1_ PES.^17,18^ Unfortunately, TS6 could not be located using single-reference DFT calculations, and as a result, dynamical calculations for this pathway were not performed. However, since the resulting H_2_ is a homonuclear diatomic molecule formed by the newly created bond of the roaming atom, its product state distributions are likely similar to those of TS4.

### 3.3 Product State Distributions

#### 3.3.1 General Features

**Figure 3:**
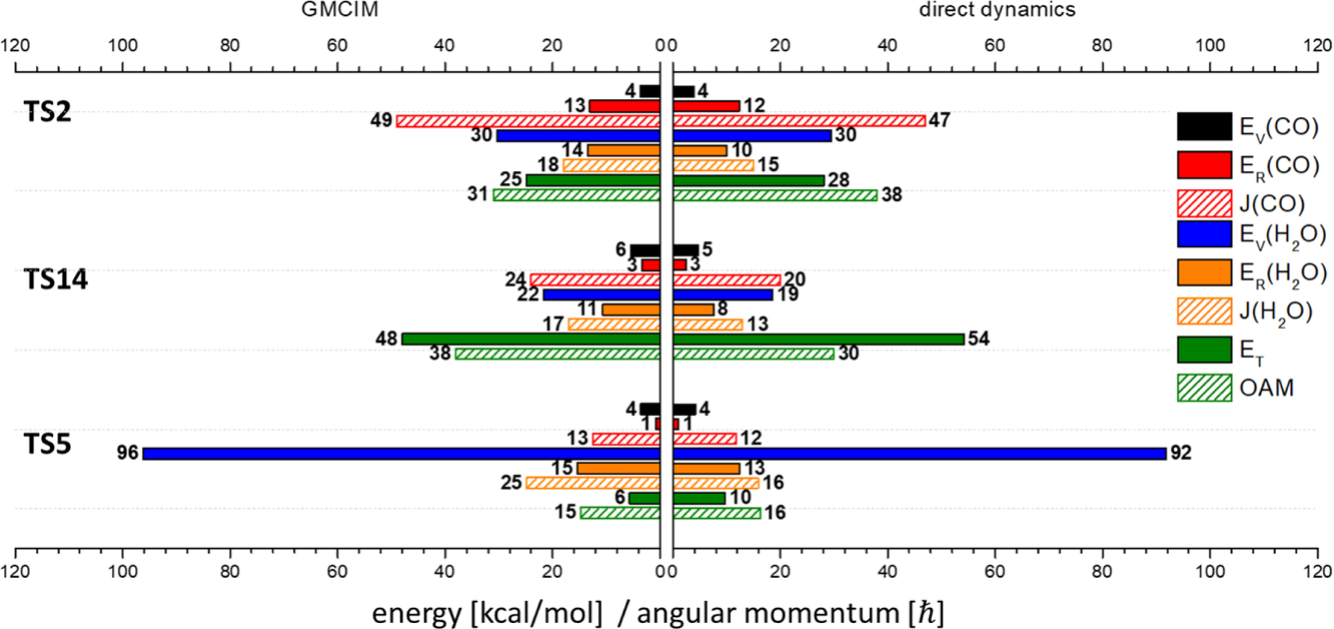
Mean values of product state distributions of CO + H_2_O pathways with ZPE of MIN1 above the barrier top calculated by direct dynamic simulation and GMCIM. The units of energy and angular momentum are in kcal/mol and ℏ, respectively.

**Figure 4:**
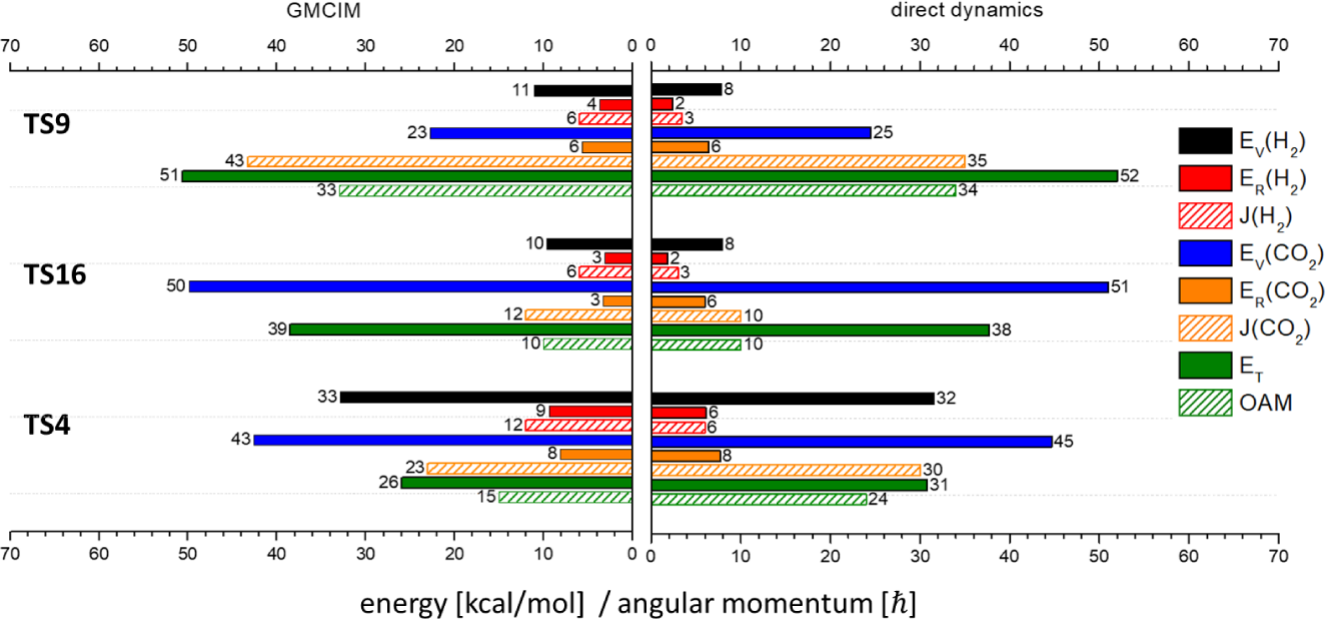
Mean values of product state distributions of CO_2_ + H_2_ pathways with ZPE of MIN1 above the barrier top calculated by direct dynamics simulation and GMCIM. The units of energy and angular momentum are in kcal/mol and ℏ, respectively.

We first present the results of the direct dynamics simulation and GMCIM for the dissociation pathways, using the ZPE of MIN1 (21.5 kcal/mol) as the initial energy above the barrier top for each dissociation pathway. At least 1000 classical trajectories for each saddle point were calculated at the M06-2X/aug-pcseg-1 level. (Tables S4 and S5) The mean values of the product-state distributions for the CO + H_2_O and CO_2_ + H_2_ channels are shown in Figures 3 and 4, respectively. Although the total energy of each pathway differed in these calculations, the general features of the product states from each dissociation pathway can be summarized as follows. The product state energies are reported in kcal/mol and include rotational energies (E_R_), vibrational energies (E_V_), and total translational energy (E_T_) of the product pairs. The classical angular momentum is reported in two components expressed in units ofℏ: the rotational angular momentum of each product, and the orbiting angular momentum (OAM) resulting from the relative motion between the product pair.

##### 3.3.1.1 CO + H_2_O Pathways

The reaction endoergicity of the CO+H_2_O dissociation channel is approximately 12 kcal/mol, as calculated at the M06-2X/aug-pcseg-1 level. At this computational level, the ZPE of CO and H_2_O are 3.3 and 13.5 kcal/mol, respectively. Considering the initial energy and the energy of each saddle point, the total energy available for the products from each pathway is calculated by subtracting the reaction endoergicity from the sum of the initial energy and the energy of each saddle point (SP): ZPE(MIN1) + E(SP) – E(products). The total energies available for the CO + H_2_O products are 84, 88, and 123 kcal/mol for the TS2, TS14, and TS5 pathways, respectively. After subtracting the ZPE of the products, the total available energies are approximately 67, 71, and 106 kcal/mol for the three pathways, respectively. Direct dynamics simulation for TS5 was initiated at a structure located 1.97(amu)^1/2^/Bohr beyond the saddle point on the MEP, approximately 2.6 kcal/mol lower in energy than the saddle point (Figure S3).

Dissociation through the TS2 pathway produces fast-moving products with non-negligible internal excitation. The CO products exhibit significant rotational excitation, while the H_2_O products are rovibrationally excited. The mean value of the rotational angular momentum of CO, J(CO), is approximately 47ℏ, corresponding to a rotational energy of 12 kcal/mol. The mean vibrational energy of H_2_O (E_V_(H_2_O)) and the total translational energy (E_T_) is 30 and 28 kcal/mol, respectively. After subtracting the ZPE of the products, the internal energy of CO, the internal energy of H_2_O, and the total translational energy account for 19%, 39%, and 42% of the total energy available for the products, respectively.

The main features of the TS14 pathway are a higher E_T_ (54 kcal/mol) and lower J(CO) (20ℏ), compared to those from the TS2 pathway. The internal energy excitation of H_2_O is also less pronounced. After subtracting the ZPE of the products, the internal energy of CO, the internal energy of H_2_O, and the total translational energy account for 6%, 18%, and 76% of the total energy available for the products, respectively.

Products from the TS5 pathway concentrate most of the total available energy into the vibrational modes of H_2_O. The remaining energy permits only limited excitation to the other degrees of freedom of the products. The rotational excitation of the CO product is similar to that of the TS14 pathway, but the mean value of the total translational energy is smaller than that of both the TS2 and TS14 pathways. After subtracting the ZPE of products, the internal energy of CO, the internal energy of H_2_O, and the total translational energy account for 2%, 89%, and 9% of the total energy available for the products, respectively.

##### 3.3.1.2 CO_2_ + H_2_ Pathways

The reaction endoergicity of the CO_2_ + H_2_ dissociation channel is approximately 4 kcal/mol, calculated at the M06-2X/aug-pcseg-1 (Table 1). At this computational level, the ZPE values for CO_2_ and H_2_ were found to be 6.5 and 6.4 kcal/mol, respectively. The total energy available for the CO_2_ + H_2_ products is calculated as 93, 105, and 123 kcal/mol for the TS9, TS16, and TS4 pathways, respectively. After subtracting the ZPE of products, the energy values are 80, 92, and 110 kcal/mol. Direct dynamics simulation of TS4 was initiated at the IRC structure located at 3.50 (amu)^1/2^/Bohr beyond the saddle point on the minimum energy path, which is 3.4 kcal/mol lower in energy than the saddle point (Figure S3).

The main features of the product states from the TS9 and TS16 pathways are similar, with a significant portion of the total available energy distributed between the translational energy (E_T_) and the vibrational energy of CO_2_, E_V_(CO_2_). The main difference between these pathways is that the vibrational excitation of CO_2_ is predominant in the TS16 pathway, while E_T_ is higher than the total internal energy of products in the TS9 pathway. The mean value of the rotational angular momentum of H_2_, J(H_2_), is approximately 3ℏfor both pathways. Although the CO_2_ products exhibit similar rotational energies in both pathways, the CO_2_ products from TS9 have a larger J(CO_2_) compared to the TS16 pathway. Since there is no adequate method to separate the contribution of internal motion (e.g., bending vibration or internal rotation) from the end-overend rotation to the angular momenta of polyatomic products in our simulations, we did not pursue this case further. After subtracting the ZPE of the products, internal energy of H_2_, the internal energy of CO_2_, and the total translational energy account for 5% (4%), 30% (55%), and 65% (41%) of the total energy available for products from the TS9(TS16) pathway, respectively.

The total available energy of the TS4 pathway is distributed among different product states. Although the vibrational excitation of the H_2_ product is more pronounced than in the TS9 and TS16 pathways, it is less pronounced than expected in typical roaming dissociation mechanisms. The vibrational energy of the CO_2_ product is higher than that of the H_2_ product, which is contrary to the typical trend observed for roaming products.^1−3,8^ Additionally, the total translational energy (31 kcal/mol) is significant, although it remains lower than that of the TS16 pathway. The rotational angular momenta of the two products do not allow differentiation between the roaming pathway and the TS9 and TS16 pathways, as the differences in rotational excitation among these pathways are not substantial. After subtracting the ZPE of the products, the internal energy of H_2_, the internal energy of CO_2_, and the total translational energy accounted for 29%, 43%, and 28% of the total energy available for the products from the TS4 pathway, respectively.

The results of the GMCIM are also shown in Figures 3 and 4 for comparison. The GMCIM simulations were performed using the same initial energy (i.e., the ZPE of MIN1) above the barrier top of each dissociation pathway. A total of 2,000 different initial conditions were randomly selected for each pathway in the simulation. Overall, the results demonstrated that GMCIM could reproduce the main features of the product states from each pathway. The mean absolute error (MAE) of the product state energies predicted by GMCIM is approximately 2 kcal/mol for all six pathways, while the MAE of angular momenta is 4 ℏ when compared with the results of the direct dynamics simulation.

#### 3.3.2 Comparison with Previous Studies at 248 nm

Kurosaki et al.^57^ investigated the product state distributions from the TS2 and TS9 pathways at 248 and 193 nm using direct dynamic simulation at the MP2 (full)/cc-pVDZ computational level. Here, we present the results of the M06-2X/aug-pcseg-1 direct dynamics simulation at 248 nm for comparison. In contrast to the CASPT2 and M06-2X calculations, the CO_2_ + H_2_ products were found to have lower energies than MIN1 on the MP2 PES. According to the MP2(full)/cc-pVDZ calculation (Table S6), the reaction endoergicity of the CO + H_2_O and CO_2_ + H_2_ channels was determined to be 10 and −4.6 kcal/mol, respectively. After including ZPE, these values were revised to 5.4 and −12.4 kcal/mol, as reported in the previous study.^57^ As previously noted, the reaction endoergicity of the CO_2_ + H_2_ channel predicted by M06-2X is +4 kcal/mol. At a given initial energy in the direct dynamics simulation, this discrepancy between the MP2 and M06-2X methods in the reaction endoergicity of the CO_2_ + H_2_ channel results in a difference of approximately 9 kcal/mol in the total available energy of the products.

The initial energies above the barrier top in our simulation at 248 nm were 62.1 and 61.0) kcal/mol for the TS2 and TS9 pathways, respectively. The mean values of the product energy distributions from TS2 and TS9 were compared with those from a previous study as follows: The total translational energy (E_T_) and the internal energies of CO and H_2_O for the TS2 pathway were 42.7, 19.5, and 59.9 kcal/mol, respectively. The corresponding values reported by Kurosaki et al.^57^ were 37.2, 26.0, and 63.4 kcal/mol. For the CO_2_ + H _2_ channel, the E_T_ and the internal energies of CO_2_ and H_2_ were 59.3, 48.2, and 25.2 kcal/mol, respectively. The corresponding values obtained by Kurosaki et al.^57^ were 69.6, 47.9, and 23.9 kcal/mol. In comparison with the previous MP2 calculations, our results showed higher E_T_ and lower internal energies of both products for the TS2 pathway. The E_T_ of the TS9 pathway that we obtained was approximately 10 kcal/mol lower than the value reported in the previous MP2 calculation,^57^ aligning with the discrepancy in reaction endoergicity between the two methods (∼9 kcal/mol).

#### 3.3.3 The Dissociation Dynamics at 230 nm

**Figure 5:**
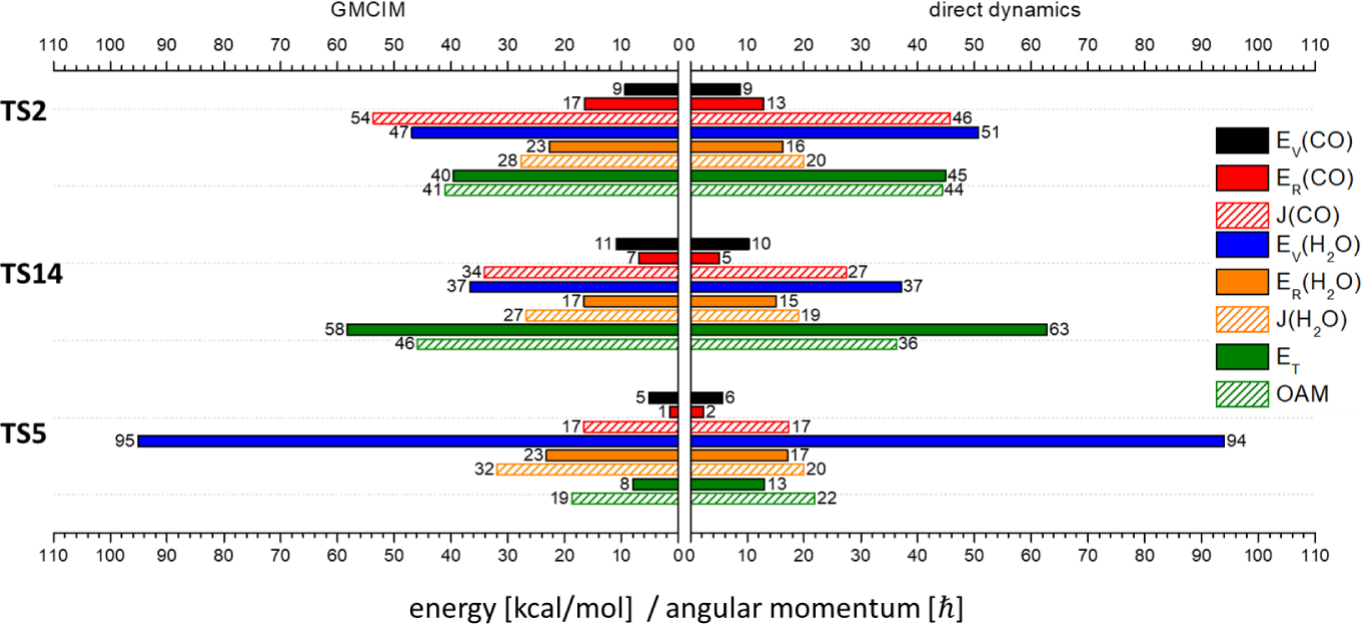
Mean values of product state distributions of CO + H_2_O pathways at 230 nm calculated by direct dynamic simulation and GMCIM. The units of energy and angular momentum are in kcal/mol and ℏ, respectively.

**Figure 6:**
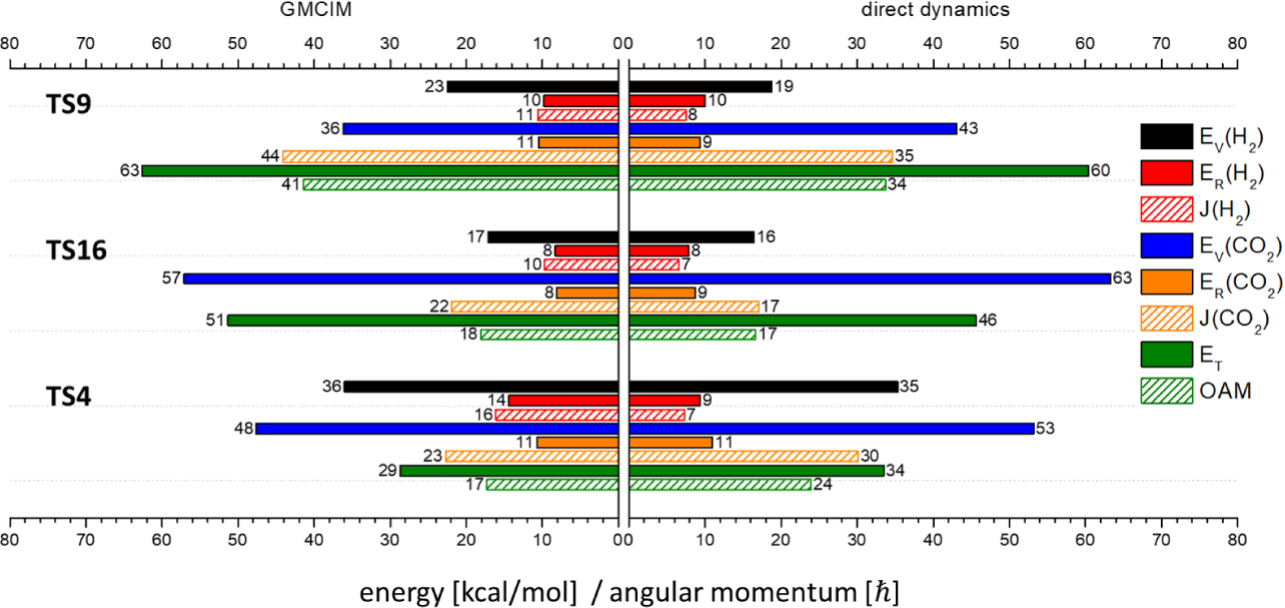
Mean values of product state distributions of CO_2_ + H_2_ pathways at 230 nm calculated by direct dynamic simulation and GMCIM. The units of energy and angular momentum are in kcal/mol and ℏ, respectively.

The mean values of the product state distributions from the direct dynamics and GMCIM simulations at a photolysis wavelength of 230 nm are summarized in Figures 5 and 6, as well as in Tables 2 and 3. Distributions of the product state energies and angular momenta from the six pathways were accumulated and displayed as histograms (Figures S4–S9), with the optimal size and number of bins chosen for clarity in each figure. The initial energies above the barrier top of each pathway were 71.2 (TS2), 67.8 (TS14), 32.8 (TS5), 70.0 (TS9), 58.8 (TS16), and 40.8 (TS4) kcal/mol, respectively. At least 2000 trajectories were simulated for each pathway (Table S4); over 98% of the trajectories from the TS2, TS9, TS14, and TS16 pathways successfully dissociated within an average time of 80 fs (Table S5). The numbers of successfully dissociated trajectories through these pathways were 5944 (TS2), 2980 (TS14), 1983 (TS9), and 1972 (TS16). The remaining 2% of the trajectories were frustrated, moving back to the reactant side without dissociating within the maximum evolution time (300 fs). For the roaming pathways (TS4 and TS5), the loose saddle point is located in the plateau region of the minimum energy path. The dissociation time and the percentage of successful trajectories depended on the starting structure along the MEP. In our simulation, the chosen starting structures yielded approximately 70% and 80% success rates for the TS5 and TS4 pathways, respectively, with average dissociation times requiring 177 and 67 fs (Table S5). The trajectories that failed to form molecular products within the maximum evolution time (400 fs) either resulted in HCO + OH or HOCO + H radical products or returned to reactants by the end of the simulation. The total number of successfully dissociated trajectories through these two roaming pathways was 5961(TS5) and 2512(TS4). (Table S4)

**Table 2:** Mean Values and Standard Deviations of the Product State Distributions of CO + H_2_O Pathways at 230 nm^a^

	TS2		TS14		TS5	
	trajectory	GMCIM	trajectory	GMCIM	trajectory	GMCIM
**E**_**V**_**(CO)**	8.8 ± 7.4	9.4 ± 7.1	10.3 ± 9.6	10.9 ± 8.7	5.7 ± 4.6	5.2 ± 2.8
**E**_**R**_**(CO)**	12.9 ± 9.2	16.5 ± 10.9	5.1 ± 4.6	6.9 ± 6.4	2.3 ± 3.7	1.47 ± 1.5
**J(CO)**	45.7 ± 17.8	53.7 ± 18.1	27.4 ± 13.7	34.1 ± 16.5	17.3 ± 11.3	16.6 ± 8.0
**E**_**V**_**(H**_**2**_**O)**	50.7 ± 20.1	46.9 ± 18.7	37.1 ± 16.2	36.7 ± 13.0	94.0 ± 15.7	95.1 ± 17.0
**E**_**R**_**(H**_**2**_**O)**	16.3 ± 10.5	22.7 ± 16.2	15.1 ± 9.7	16.6 ± 12.7	17.1 ± 11.8	23.2 ± 16.4
**J(H**_**2**_**O)**	20.0 ± 7.7	27.7 ± 10.7	19.0 ± 7.0	26.7 ± 8.8	20.0 ± 6.7	31.9 ± 11.7
**E**_**T**_	44.9 ± 16.2	39.6± 16.0	62.8 ± 15.3	58.3 ± 13.3	13.0 ± 7.4	8.0 ± 4.2
**OAM**	44.3 ± 19.2	41.0 ± 19.9	36.2 ± 16.8	45.9 ± 21.6	21.9 ± 13.0	18.7 ± 10.9

a The units of energy and angular momentum are in kcal/mol and ℏ, respectively

**Table 3:** Mean Values and Standard Deviations of the Product State Distributions of CO_2_ + H_2_ Pathways at 230 nm^a^

	TS9		TS16		TS4	
	trajectory	GMCIM	trajectory	GMCIM	trajectory	GMCIM
**E**_**V**_**(H**_**2**_**)**	18.2 ± 14.4	22.5 ± 12.1	16.4 ± 11.3	17.1 ± 9.9	35.3 ± 19.0	36.0 ± 13.4
**E**_**R**_**(H**_**2**_**)**	10.0 ± 9.7	9.8 ± 9.0	7.9 ± 8.1	8.3 ± 7.1	9.4 ± 9.6	14.4 ± 12.3
**J(H**_**2**_**)**	7.5 ± 4.1	10.6 ± 5.2	6.6 ± 3.7	9.7 ± 4.7	7.3 ± 3.8	16.1 ± 7.5
**E**_**V**_**(CO**_**2**_**)**	43.1 ± 16.8	36.2 ± 16.0	63.3 ± 18.3	57.1 ± 17.3	53.2 ± 18.4	47.6 ± 16.6
**E**_**R**_**(CO**_**2**_**)**	9.4 ± 8.4	10.5 ± 9.6	8.7 ± 8.5	8.1 ± 7.8	11.0 ± 9.1	10.7 ± 10.2
**J(CO**_**2**_**)**	34.6 ± 14.0	44.1 ± 15.9	17.0 ± 8.0	21.9 ± 9.0	30.1 ± 10.2	22.7 ± 9.2
**E**_**T**_	60.4 ± 17.1	62.6 ± 16.5	45.6 ± 14.9	51.3 ± 17.2	33.5 ± 13.3	28.7 ± 11.8
**OAM**	33.7 ± 14.7	41.4 ± 18.2	16.6 ± 8.6	18.1 ± 9.5	23.9 ± 9.7	17.3 ± 9.4

a The units of energy and angular momentum are in kcal/mol and ℏ, respectively

The main features of the product state energies and angular momenta from these pathways at 230 nm were similar to the corresponding outcomes at the initial energy of the zero-point energy (ZPE) of MIN1. However, unlike the simulations at the ZPE, the total energies of the simulations at 230 nm were consistent across different dissociation pathways. The increase in initial energy was pathway-dependent when the simulations were switched from the ZPE of MIN1 to 230 nm photolysis. Consequently, the increase in the product energies due to the higher initial energy was also pathway-dependent. Pathways with lower barrier heights such as TS2 and TS9, exhibited significant changes in product states when comparing the simulated results at 230 nm with those at the ZPE. In contrast, the two roaming pathways (TS5 and TS4), which have higher barrier heights, showed product energies at 230 nm that were more similar to those at the ZPE.

##### 3.3.3.1 CO + H_2_O Pathways

The total energy available for CO + H_2_O products at 230 nm was approximately 133 kcal/mol. After subtracting the ZPE of the products, the total available energy was determined to be approximately 116 kcal/mol. Dissociation through the TS2 pathway produced fast-moving products with non-negligible internal excitation. The mean value of J(CO) was approximately 46ℏ, corresponding to a rotational energy of 13 kcal/mol. The mean vibrational energy of H_2_O and the total translational energy were 51 and 45 kcal/mol, respectively. After subtracting the ZPE of the products, the internal energies of CO and H_2_O, as well as the total translational energy (E_T_) accounted for 16%, 46%, and 38% of the total energy available for the products, respectively.

The products from the TS14 pathway had mean values of E_T_ and J(CO) of 63 kcal/mol and 27ℏ, respectively. After subtracting the ZPE of the products, the internal energies of CO, the internal energy of H_2_O, and E_T_ accounted for 11%, 34%, and 55% of the total energy available for the products, respectively.

The products from the TS5 pathway accumulated large amounts of energy in the vibrational mode of H_2_O. After subtracting the ZPE of the products, the internal energies of CO, H_2_O, and E_T_ accounted for 4%, 85%, and 11% of the total energy available for the products, respectively.

##### 3.3.3.2 CO_2_ + H_2_ Pathways

The total energy available for CO_2_ + H_2_ products under 230 nm photolysis was 142 kcal/mol at the computational level of M06-2X/aug-pcseg-1. The corresponding energy value after subtracting the ZPE of the products was approximately 129 kcal/mol.

The main features of the product states from TS9 and TS16 were similar at 230 nm, except that the products from the TS16 pathway exhibited higher vibrational excitation of CO_2_, whereas the products from the TS9 pathway had higher translational energy. The mean values of J(H_2_) were approximately 7–8 ℏ for both pathways. The CO_2_ products from TS9 exhibited larger J(CO_2_) values compared to those from TS16. After subtracting the ZPE of the products, the internal energies of H_2_, CO_2_, and E_T_ account for 17% (14%), 36% (51%), and 47% (35%) of the total energy available for the products from the TS9 (TS16) pathway, respectively. The main feature of the product state energies from the TS4 pathway at 230 nm was similar to that observed at the initial energy of the ZPE. Both the translational and internal energies of the products were excited by the potential energy released from the exit barrier in the dissociation pathway. After subtracting the ZPE of the products, the internal energies of H_2_, CO_2_, and E_T_ account for 30%, 44%, and 26% of the total energy available for the products from the TS4 pathway, respectively.

The GMCIM simulation was performed by considering the same initial energy of 230 nm photolysis, with 10,000 different initial conditions randomly selected for each pathway. The mean absolute error (MAE) of the product state energies and angular momenta predicted by GMCIM was approximately 3 kcal/mol and 8ℏ for the six pathways, respectively, when compared with the results of the direct dynamic simulation. Larger absolute errors in the predictions vibrational energies for polyatomic products and the total translational energies were observed. The larger errors in angular momentum were associated with the roaming pathways (TS4 and TS5).

The changes in product states with the increase in initial energy from ZPE to 230 nm photolysis are summarized as follows: The total translational energies of the roaming pathways (TS5 and TS4) changed only slightly, with the increase in the initial energy contributing primarily to the internal energies of the products. For the TS2 and TS14 pathways, both the internal energies of H_2_O and the total translational energies increased significantly. In the TS9 and TS16 pathways, the increase in the internal energy of CO_2_ was the most prominent feature.

#### 3.3.4 Comparisons with Experimental Results at 230 nm

**Figure 7:**
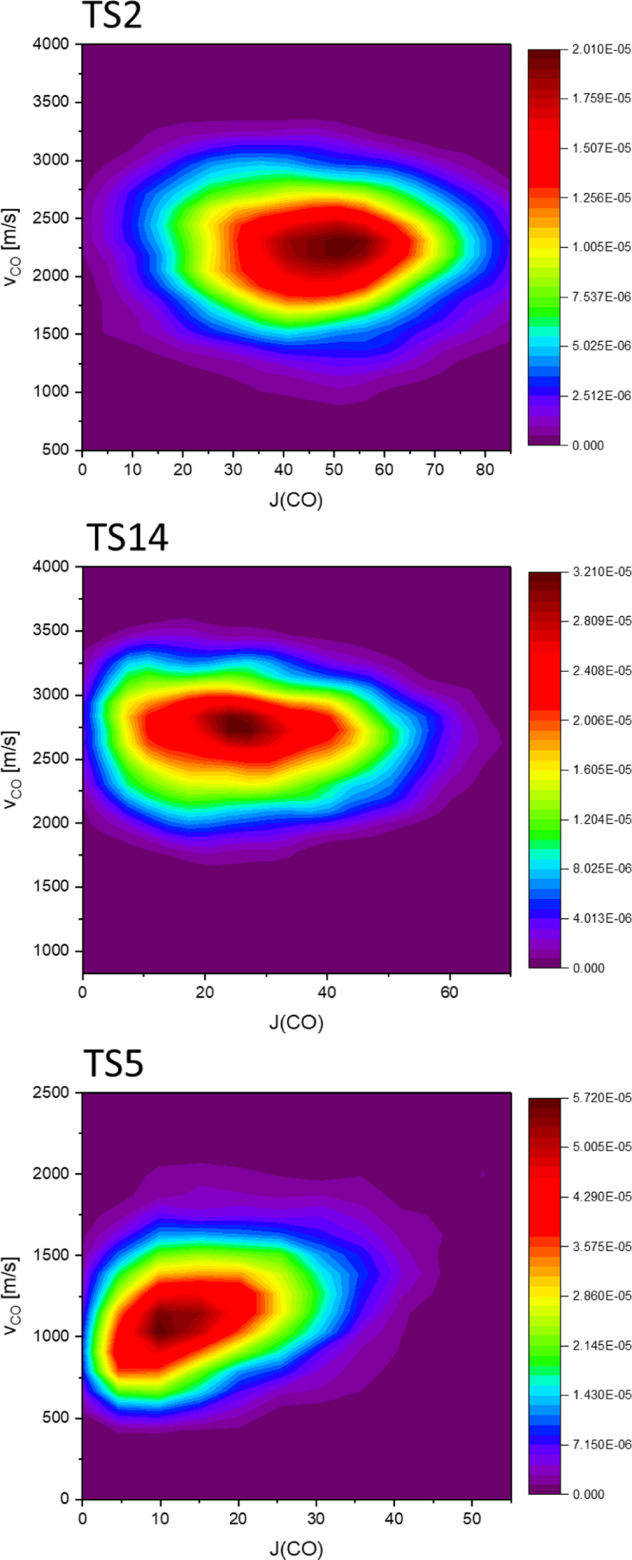
v_CO_ vs J(CO) correlation diagrams of TS2, TS14 and TS5 pathways. The most probable speed of CO (v_CO_) is almost unchanged with respect to angular momentum of CO (J(CO)) in TS2 and TS14 pathways. The most probable v_CO_ slightly increases with the increasing of J(CO) in TS5 pathway.

To provide a detailed comparison with the state-resolved experimental results of CO products,^16^ it would be advantageous to examine the state-resolved speed distribution of the simulations. However, due to the limited number of trajectories in our direct dynamic simulation, it was challenging to obtain reliable state-resolved information using Gaussian or histogram binning procedures. Therefore, we did not perform a binning procedure for state selection but instead provided kernel density plots for the two-dimensional correlation diagrams of the two types of product states. For example, the J_CO_ vs v_CO_ diagrams for the three CO-forming pathways (TS2, TS14, and TS5) are shown in Figure 7. Before preparing these plots, trajectory events in which the vibrational energies of the CO products were below their ZPE were excluded. After removing trajectories with E_V_(CO) < ZPE(CO), the remaining numbers of trajectories for the TS2, TS14, and TS5 pathways were 4142, 2212, and 3970, respectively(Table S4).We observed that the most probable value of the velocity distribution of the CO product was almost independent of its vibrational energy (Figure S10). Therefore, to ensure sufficient trajectory data, we did not further select CO vibrational states from the direct dynamics results. The J_CO_ vs v_CO_ correlation diagram (Figure 7) provides qualitative features of J(CO)-state-resolved product speed distributions, allowing us to interpret the trend of the v_CO_ distribution as a function of J(CO) as observed in the ion-imaging experiment at 230 nm.^16^ The J(CO) distribution of products from the TS2 pathway exhibits a normal distribution, with the peak value located at J(CO) ∼ 45ℏ. For J(CO) varying from 20 to 50ℏ, the most probable values of v_CO_ from the TS2 pathway (∼2300 m/s) remained constant with respect to J(CO). Although the peak positions of the v_CO_ distributions slightly increased to approximately 2500 m/s at low J (CO) (<10ℏ), the relative population in such J(CO) states was minimal (Figure 7). From the J(CO) versus v_CO_ diagram of the TS14 pathway, the most probable J(CO) was approximately 27 ℏ, and the peak positions of v_CO_ (2750 m/s) remained independent of J(CO). In contrast, the most probable value of v_CO_ exhibited a positive correlation with J(CO) in the TS5 pathway. The peak value of v_CO_ was 900 m/s for J(CO) < 10ℏ, increasing to 1300 m/s when J(CO) ∼ 20ℏ. According to a previous study,^66^ GMCIM may fail to reproduce the two-dimensional correlation of product states, such as the J(CO) vs E_T_ correlation of the TS pathway in acetaldehyde. Therefore, for comparison with the experiment, we selected only the vibrational state of v(CO) = 0 for the GMCIM results without further selection of J(CO).

In a previous one-color experiment,^16^ time-sliced ion velocity imaging at 230 nm was used to study the neutral CO(X^1^Σ^+^, v = 0, J) products. These products were ionized via (2 + 1) resonance-enhanced multiphoton ionization (REMPI) through the CO (B^1^Σ^+^, v = 0, J) intermediate states. The velocity distributions of state-specific CO products were measured at J(CO) = 9, 20, 30, and 48. The photolysis energies for detecting different J(CO) states of the CO products were nearly identical (<0.1 kcal/mol).^16^ Therefore, differences in the total energies. The experimental observations are summarized as follows:

1. The observed velocity images were isotropic, indicating that the products were generated via dissociation channels of the electronic ground state of formic acid. Speed distributions corresponding to different positions of the Doppler profiles were obtained by integrating the signals on the sliced images with small angular ranges to account for contributions from nonresonant background CO^+^ species.^16^

2. The state-specific speed distributions of the CO products exhibited bimodal features at low rotational states (J(CO) = 9 and 20). An intense peak was observed at a slow speed (∼400 m/s), and another peak appeared at a fast speed (∼1300 m/s) in the speed distribution for J(CO)= 9. A weak peak at 400 m/s and another intense peak at 1500 m/s were observed for J(CO) = 20.

The results for the higher J(CO) states (30 and 48) were influenced by a strong background signal,^16^ leading to the reporting of two peak values for each J(CO) state. The background from nonresonant CO^+^ ions resulted in a peak at 1150 m/s in the speed distributions for J(CO) = 30 and 48, whereas the CO REMPI signals indicated a most probable speed of approximately 2200 m/s for both J(CO) states.

**Figure 8:**
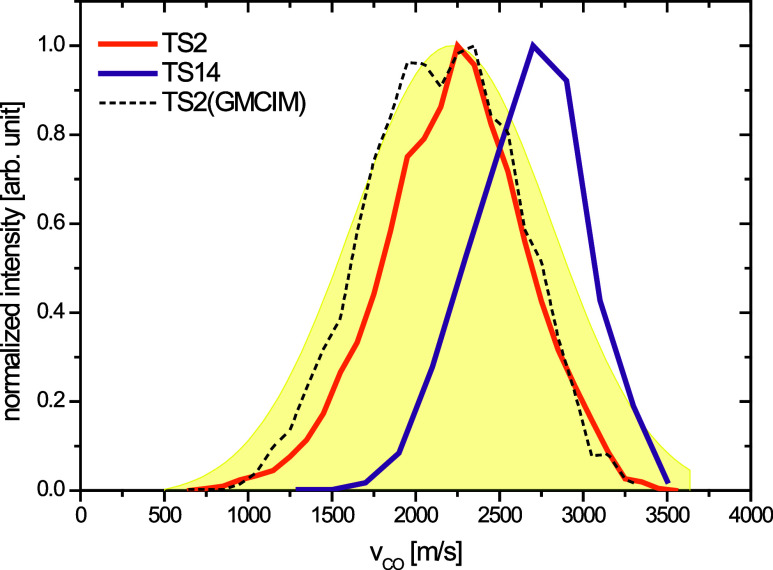
Speed distributions of CO from TS2 and TS14 pathways, without the rovibrational state-selections of CO products. The yellow shaded area is experimental result of the fast component of J (CO) = 30 speed distribution, it is the tracing of Figure 5b of ref (16). Reproduced from ref (16). Copyright 2019 American Chemical Society.

**Figure 9:**
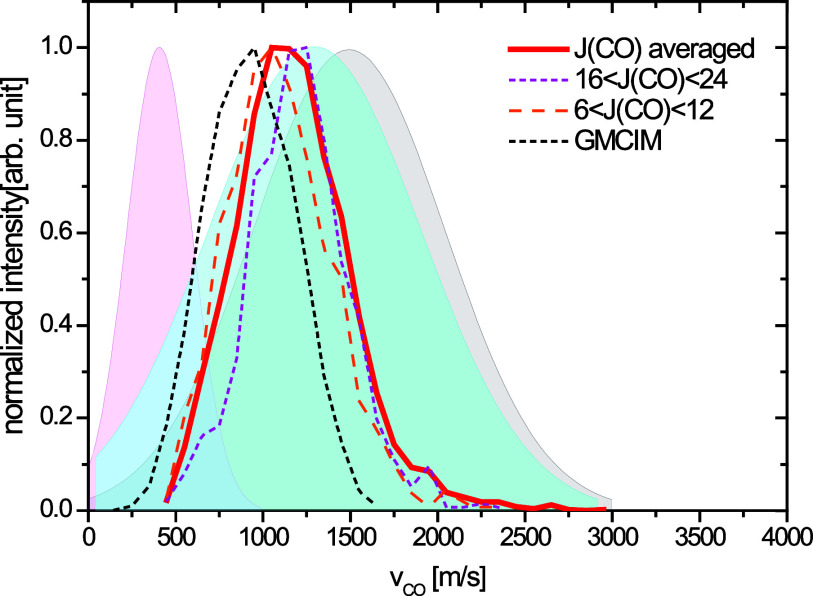
Speed distributions of CO from TS5 pathway, with or without the rotational state-selections of CO products. The blue and gray shaded area are experimental results of the fast components of speed distributions from J (CO) = 9 and 20, respectively. The pink shaded area is the experimental result of slow component of speed distribution (J(CO) = 9). The experimental results are the tracing of Figure 5a,b of ref (16). Reproduced from ref (16). Copyright 2019 American Chemical Society.

The speed distribution of the CO product can be derived from the simulated E_T_ distribution using the energy and momentum conservation laws of two-body dissociation:
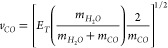


By employing this relationship, the computational data for the TS2, TS14, and TS5 pathways were compared with the experimental speed distributions of the CO products. The experimental results for J (CO) = 30ℏ, corresponding to Figure 5b in ref (16), were merged with the computational results from the TS2 and TS14 pathways, as shown in Figure 8. All computational data presented were derived from a histogram with a binning size of 100 m/s. The speed of the CO product from the TS14 pathway was found to be excessively high, with its peak value approximately 500 m/s greater than that of the experimental observation^16^ (∼2200 m/s). The results for the TS2 pathway align more closely with the experimental speed distribution of higher rotational states (J (CO) = 30 and 48ℏ). The experimental speed distributions of the low rotational states (J(CO) = 9 and 20ℏ) were compared with their computational counterparts from the TS5 roaming pathway. In Figure 9, our results are merged with the tracing of both the slow and fast components of the experimental speed distributions J (CO) = 9 and 20 ℏ, as shown in Figure 5ab of ref (16), respectively). The TS5 pathway result exhibits better agreement with the fast components of J (CO) = 9 and 20 ℏfrom the experiment, rather than with the slow component located in the lower speed region (∼400 m/s). Although CO products from the TS14 pathway show significant populations around J(CO) = 9 and 20 ℏ, their speeds are much higher than the experimental results and are therefore excluded from Figure 9. Due to the limited number of trajectories a coarse selection of J (CO) states was made for the computational results from the TS5 (Figure 9) and TS2 (Figure S11) pathways. The outcomes did not change significantly after state selection and did not enhance the comparison with experimental results. For the TS5 pathway, the peak value of v_CO_ increased with J (CO); however, the entire profile was approximately 250 m/s slower than the experimental results, as shown in Figure 9. We also present the v (CO) = 0, J(CO)-averaged results of GMCIM in Figures 8 and 9 by selecting those events where the vibrational energy of CO lies between the ZPE (3.3 kcal/mol) and the v = 1 vibrational state (9.9 kcal/mol) at the M06-2X/aug-pcseg-1. Although GMCIM predicts a slower v_CO_ for products from the TS5 pathway (approximately 150 m/s lower), its prediction of the v_CO_ of the product from the TS2 pathway aligns well with both the experimental and direct dynamics simulation results.

## 4 Discussion

### 4.1 General Features of the Product State Distributions

This study provides detailed insights into the product state features from various pathways of the CO + H_2_O and CO_2_ + H_2_ dissociation channels. As highlighted in the results, each dissociation pathway exhibited unique product-state distributions at a photolysis wavelength of 230 nm. GMCIM demonstrated comparable performance in predicting the dissociation dynamics of formic acid, akin to its effectiveness in prior studies on methyl formate and acetaldehyde.^61,66,74^ In comparison with direct dynamics simulation, GMCIM predicts the general product states features for each pathway, suggesting that the distribution can be rationalized based on the nuclear gradient along the dissociation path and the 3N–7 transverse modes.^60,61^ In addition, final state interaction plays minor role in the dissociation dynamics of these pathways.

While TS2 and TS9 were previously examined using direct dynamics simulations,^57^ this study analyzed the dynamic features of TS14, TS16, and the two roaming pathways (TS5 and TS4) for the first time. For the CO + H_2_O channel, TS2 is a typical three-center saddle point that provides higher J(CO) excitation compared to TS14 and TS5. TS14 is a four-center saddle point, and the products from this pathway exhibit higher translational energy, compensated for by the lower internal state excitation of E_V_(H_2_O) and J(CO). The difference between the TS2 and TS14 pathways can be attributed to the dynamic nature of the three-center and four-center saddle points. CO products from a carbonyl group can have higher rotational excitation^61^ by following a three-center dissociation pathway, such as TS2. The TS5 pathway shows typical roaming signatures in its product state distributions; the vibrational states of H_2_O are highly excited, leaving a limited amount of residual energy for the other degrees of freedom. These features are similar to those found in roaming dissociation mechanisms of carbonyl-containing molecules.^1−3,8,15^ Previous studies^60,61,66^ have shown that such characteristics of the roaming mechanism can be ascribed to the direction of gradient vectors during the rapid potential energy drop along the dissociation coordinate, before the formation of final products. As shown in Figure S2, the IRC profile of the TS5 pathway reveals a region with a rapid drop in potential energy. During this step, the H atom on the HCO moiety approaches the OH moiety, with the centers of mass of the two moieties remaining stationary during the decrease in potential energy. This suggests that the direction of the gradient vectors primarily contributes to the relative vibrational motion of H–OH. As the H–OH distance approaches the equilibrium bond length of the H_2_O product, the potential energy shows a gentle decline (Figure S2). At this stage, the center-of-mass (COM) of H_2_O and CO start to separate from each other along the IRC, corresponding to a minor excitation of the translational energy.

For the CO_2_ + H_2_ products, both TS9 and TS16 are four-center saddle points; therefore, the rotational excitations of the products are limited. The impulse is exerted on two atoms of a product molecule, making it difficult to generate significant torque during dissociation. The CO_2_ products from the TS16 pathway exhibit low rotational excitation because the gradients along the dissociation pathway are evenly distributed across both oxygen atoms of CO_2_. Therefore, molecules can only rely on the torque contributed by the transverse vibrations for the rotational excitation of CO_2_ product.

As far as the roaming dissociation mechanism is concerned, the product state distributions from TS4 pathway are rather peculiar. Approximately 24% of the total product energy contributes to the translational motion of products, and both products exhibit significant internal state excitation. As shown in Figure S2, these features can be explained by the direction of the gradient vectors along the IRC. In the region where the potential energy undergoes a rapid drop, half of the energy corresponds to the approach of the H--H distance, while the other half contributes to the COM separation of the CO_2_ and H_2_ moieties. Therefore, the energy of the exit barrier of the TS4 pathway does not solely flow into the vibrational mode of H_2_.

### 4.2 Comparison with a Previous Computational Study at 248 nm

We compared our results for the TS2 and TS9 pathways at 248 nm with a previous computational study^57^ conducted at the MP2 (full)/cc-pVDZ level. Due to the differences in the electronic structure calculation methods, discrepancies were observed in the product state distributions, particularly for the TS9 pathway. There was approximately a 10 kcal/mol difference in the total product energies of the two sets of TS9 data, with a similar difference observed in their total translational energies. This discrepancy can be attributed to the shape of the PES described by the different electronic structure methods. Table S6 summarizes the relative energies of the stationary points in the TS2 and TS9 pathways, obtained using the M06-2X and MP2 methods. The ZPE of stationary points was considered for comparison with the reference values.^57^ For the TS9 pathway, MP2 predicts the exoergicity of the products (−4.6 kcal/mol without including ZPE), whereas the results of both M06-2X and MS-CASPT2^17^ reveal an endoergic feature instead (+4.0 and +4.2 kcal/mol, respectively). A net difference of 8.6 kcal/mol in reaction endoergicity between the M06-2X and MP2 calculation leads to the same difference in the available energy of product. The exit barrier height, defined as the energetic difference between the saddle point and the product, shows a net difference of 4.5 kcal/mol between the M06-2X and MP2 calculation (without including ZPE). The energy of the exit barrier is primarily assumed to contribute to the translational energy of products,^1,2,8,25^ particularly in pathways that generate fast-moving products such as TS9. Consequently, when comparing with M06-2X calculation, the exoergicity of CO2+H2 predicted by the MP2 calculation leads to a higher exit barrier height and available energy of product, results in larger translational energy. This assumption explains the discrepancy between the results of this study and those of a previous computational study at 248 nm.^57^

### 4.3 Comparisons with Experimental Results at 230 nm

A previous experimental study at 230 nm reported an isotropic angular distribution of CO products, implying that the experimentally observed CO products were likely formed via dissociation pathways on the ground state PES of formic acid.^16^ The isotropic angular distribution enables the deconvolution of the REMPI signal of neutral CO products from the strong CO^+^ ion background by distinguishing their distinct Doppler shifts,^16^ facilitating the comparison of experimental and computational results. The speed distributions of the CO products (v_CO_) from the TS2, TS14, and TS5 pathways at 230 nm derived from both direct dynamics and GMCIM simulations, were compared with the experimental results (Figures 8 and 9). This comparison highlights several features that not only elucidate the experimental observation but also provide deeper insight into the dissociation mechanism of formic acid at 230 nm. These features are summarized as follows:

#### 4.3.1 The Contribution of TS14 Pathway to CO Production is Negligible

The J(CO) distribution of products from the TS14 pathway is broad (J(CO) = 0–60ℏ) (Figure S5). Therefore, if the experimental products originated from the TS14 pathway, they would appear in both high J(CO) and low J(CO) states. Computational results for the speed distribution show a peak value exceeding 2500 m/s, remaining almost independent of J(CO). Hence, if the TS14 pathway significantly contributed to the experimental results, one would expect to observe fast-moving CO (v_CO_ > 2500 m/s) across all J(CO) states. However, this is not supported by the experimental speed distributions,^16^ where the majority of CO speeds at different J(CO) values were below 2500 m/s.^16^ Therefore, it can be concluded that the TS14 pathway is not the dissociation pathway observed in the experiment at 230 nm, suggesting that isomerization (MIN1 **→** MIN4) before dissociation is unlikely.

#### 4.3.2 TS2 is the Predominant Pathway for Generating High J(CO) Products (J(CO) ≥ 30ℏ)

According to the results of the direct dynamics simulation at 230 nm (Figure S4), the J (CO) distribution of the TS2 pathway was approximately normally distributed with a symmetric shape. The peak value of J(CO) was approximately 45 ℏ, and the population decreased dramatically significantly for J(CO) < 20 ℏ. If this pathway is observed in the experiment at 230 nm, it would primarily appear in the speed distributions of J(CO)=30 and 48ℏ, with a minor contribution to J(CO) = 20 ℏ. The computational speed distribution of TS2 had a peak value around 2200 m/s and was almost independent of J(CO). Figure 8 demonstrates that the speed distribution of the TS2 pathway aligns well with the fast component observed in the high-J (CO) states (J(CO) = 30 ℏ). For J(CO) = 20 ℏ, the high-speed tail of the experimental distribution (∼2200 m/s) is also covered by the computational results of TS2. Therefore, it can be concluded that the TS2 pathway likely corresponds to the dissociation mechanism observed experimentally in the high-J (CO) states at 230 nm.

#### 4.3.3 TS5 Contributes Significantly to the Low J(CO) Products (J(CO) ≤ 20ℏ)

The J (CO) distribution of the products from the TS5 pathway exhibited an asymmetric shape, with a mean value of 17 ℏ at 230 nm (Table 2). The intensity of the distribution dropped to approximately one-third of the peak value when J(CO) > 30 ℏ (Figure S6). Thus, if the products from the TS5 pathway were observed in the experiment, they would predominantly contribute to the J(CO) < 30 ℏ. Furthermore, the nonresonant background of CO^+^ can interfere with the low-speed region of the experimental speed distribution at high J(CO) states.^16^ As a result, the contribution of the TS5 pathway to products with J (CO) > 30 ℏ cannot be definitely determined. The results of the direct dynamics simulation indicate that the speed distribution of the TS5 pathway peaks at approximately 1100–1300 m/s (Figure 7), depending on the J(CO) states. The fast components in the experimental speed distributions for J(CO) = 9 ℏ and 20 ℏwere located at 1300 and 1500 m/s, respectively. Therefore, it is more reasonable to attribute the fast component (1300–1500 m/s) to the roaming pathway (TS5) rather than the slow component (∼400 m/s) in the experimental speed distributions at low J(CO) states. As suggested in a previous study, the slow component may be associated with the presence of clusters in the molecular beam.^16^ Another possible explanation is the secondary dissociation of HCO from the barrierless HCO + OH pathway on the S_0_ surface, although this is less likely to occur at 230 nm, as previously proposed.^16^ The findings of this study provide theoretical evidence to rule out the relevance of the TS5 pathway to the slow component of the experimental speed distribution in low-J (CO) states.

### 4.4 The Dissociation Mechanism at 230 nm

A previous theoretical study^17^ conducted a comprehensive investigation into the potential energy surfaces of the ground (S_0_) and excited electronic states (S_1_, and T_1_) of formic acid. The dissociation pathways of all significant dissociation channels were identified through a systematic search of the dissociation saddle points and minimum energy paths. Based on these findings, dissociation mechanisms of various channels at different photolysis energies have been proposed, including an explanation of conformational memory at 193 nm.^17,18,48^ In this study, we focus solely on the dissociation pathways of CO + H_2_O channel at 230 nm, aiming to integrate the previously proposed dissociation mechanism with the dynamical information revealed in this work. The HCO + OH channel predominantly occurs at photolysis energies above 119 kcal/mol (240 nm) due to the opening of the direct dissociation pathway on the S_1_ surface.^17,41^ While the OH-forming channel is expected to dominate at 230 nm, it is reasonable to assume that the quantum yield of OH production at this wavelength does not exceed its value at 222 nm (∼0.8).^41,51^ Consequently, as long as nonradiative decay allows molecules to relax back to the S_0_ state, the CO + H_2_O channel may serve as one of the minor products at 230 nm. A previous study proposed various nonradiative decay pathways at different available energies. Here, we considered only those pathways that did not involve the S_2_ state, as the available energy is below 148 kcal/mol (193 nm).

To specifically describe these radiationless decay pathways, we outline the dissociation pathways of the three radical channels on the S_1_ and T_1_ excited state PESs. Terms and labels from a previous study^17^ were adopted to denote key stationary points, including the saddle points of the dissociation pathways, the minimum energy conical intersections (MECIs) between the S_0_ and S_1_ states, and the minima on the seam of crossing (MSX) between the singlet and triplet states. The MECIs and MSXs on the PESs represent configurations that facilitate nonradiative decay. The three radical channels are as follows:

(a) C–H bond breaking channel (H + COOH). The saddle points on the T_1_ surface are T_1_–TS4 (cis-COOH) and T_1_–TS7 (trans-COOH) The saddle points on the S_1_ surface are inaccessible under 230 nm photolysis and are therefore omitted here.^17^

(b) OH-forming channels (HCO + OH). The saddle points on the S_1_ and T_1_ surfaces are S_1_–TS2 (117.4 kcal/mol) and T_1_–TS3(114.8 kcal/mol), respectively.^17^

(c) O–H bond breaking channel (HCOO + H). The saddle points on both the S_1_ and T_1_ surfaces are inaccessible under 230 nm photolysis and are not considered further in discussion. The experimental threshold for this channel was determined to be 127.2 kcal/mol (∼225 nm).^17,45^

Based on this information, four distinct nonradiative decay paths originating from the S_1_ surface are energetically accessible at a photolysis energy of 230 nm:^17^

(i) S_1_/T_1_ Intersystem Crossing (ISC): The molecule undergoes ISC at the minimum of the S_1_ surface before encountering any barriers of the radical channels, followed by T_1_/S_0_ ISC to the S_0_ state via S_0_/T_1_–MSX5 or S_0_/T_1_–MSX6 on the T_1_ surface. There are no significantly elongated bonds in the geometries of these MSXs (see Figure 1 of ref (17)); consequently, no such features exist after relaxation to the S_0_ surface. This decay path becomes energetically accessible when the available energy exceeds 110.4 kcal/mol (∼259 nm).^17,42−44^

(ii) Molecules relax from the S_1_ to the T_1_ state via ISC, similar to path (i). On the T_1_ surface, C–OH bond elongates to cross T_1_–TS3 in channel (b), followed by ISC to the S_0_ state via S_0_/T_1_–MSX4.^17^ The geometry of this MSX resembles a partially dissociated HCO + OH,^17^ analogous to the TS5 saddle point. This decay path becomes accessible when the available energy surpasses the energy^17^ of T_1_–TS3, which is 114.8 kcal/mol (∼249 nm).

(iii) Molecules undergo ISC from S_1_ to T_1_ in the same manner as in path (ii), but instead elongate the C–H bond to cross T_1_–TS4 or T_1_–TS7 in channel (a) Subsequently, ISC occurs to the S_0_ state via S_0_/T_1_–MSX3.^17^ The geometry of this MSX resembles a partially dissociated H–COOH, akin to the TS4 saddle point. This decay path becomes accessible when the available energy exceeds the energy^17^ of T_1_–TS7, which is 115.1 kcal/mol (∼248.5 nm).

(iv) After elongating the C–OH bond and crossing the barrier top (S_1_–TS2) of the radical channel (b) on the S_1_ surface, the molecules may undergo internal conversion (IC) to S_0_ via S_0_/S_1_–MECI1 or transition through successive ISC via S_1_/T_1_–MSX1 and S_0_/T_1_–MSX4 to the S_0_ state. Similar to S_0_/T_1_–MSX4, the geometries of these MECIs and MSXs resemble those of TS5. This decay path becomes accessible when the available energy exceeds the energy^17^ of S_1_–TS2, which is 117.4 kcal/mol (∼243.5 nm).

All four decay paths listed above are energetically accessible at 230 nm. However, it is conventionally suggested that path (iv) ispredominant,^16,51^ because the S_1_–TS2 pathway of channel (b) is recognized as the major dissociating route at 230 nm.^16,51^ Although a previous study^51^ on the ion images of OH radicals suggests that the major pathway of the OH-forming channel at 230 nm occurs on the S_1_ PES, the total translational energy of OH + HCO at 230 nm spans a wide range from 0 to 17 kcal/mol (see Figure 5 of ref (51)). The broad translational energy distribution at 230 nm encompasses the range observed at 244 nm,^51^ suggesting that the possibilities of internal conversion (IC) and intersystem crossing (ISC) decay paths (i.e., path (i)–(iii)), followed by dissociation pathways on the S_0_ or T_1_ PESs, cannot be excluded.^51^ Consequently, it is not possible to eliminate any of the four decay paths from consideration, and the current results cannot definitively clarify the specific nonradiative decay path involved in the experimental observations.

Molecules may undergo a radiationless transition to the S_0_ surface through one of these nonradiative decay paths, and the resulting molecular geometries can depend on the specific path taken. Different decay paths can bring the molecules to different regions of the S_0_ surface, potentially leading to varying dissociation pathways. Unfortunately, the correlation between dissociation pathways and nonradiative decay paths preceding dissociation remains unclear. For instance, molecules following decay path (ii) may have geometries resembling the TS5 saddle point; however, this does not necessarily indicate they will dissociate into CO + H_2_O product via the TS5 pathway. Instead, the molecular flux may be directed to other dissociation pathways depending on the instantaneous mechanical states of the molecules and the local gradients on the S_0_ PES. The findings of this study do not clarify the correlation between nonradiative decay paths and dissociation pathways but focus on characterizing the features of the product states from each dissociation pathway. In other words, while the results effectively distinguish products originating from different dissociation pathways, they do not reveal which nonradiative decay path the molecules follow before entering a specific dissociation pathway. Gaining such insights would require, nonadiabatic dynamic simulation, which falls outside, the scope of this research.

### 4.5 Detection of the CO_2_ + H_2_ Channel

To date, there has been no experimental evidence identifying the dissociation pathway(s) of the CO_2_ + H_2_ products through the analysis of product state distributions. The findings of this study may offer valuable guidance for such investigations. According to the results, the strategy for distinguishing the formation pathways of the CO_2_ + H_2_ product can be summarized as follows: The CO_2_ products from the TS9, TS16, and TS4 pathways exhibit significant rovibrational excitations, making it challenging to identify the dissociation pathways solely based on the infrared emission spectra of the CO_2_ products. A more promising approach might involve measuring the speed distribution of state-specific H_2_ products, as each pathway displays distinct combinations of E_T_ and E_V_(H_2_)/E_R_(H_2_). (Figures S7–S9) Selecting rovibrational state-specific H_2_ products for speed measurements is a feasible approach.^7^ These measurements could offer valuable insights into the detailed mechanism of conformational memory in the photolysis of formic acid.^17,18,48,54^ As noted earlier, the product state distribution of the TS6 pathway remains undetermined in this study. The features of its product states may either resemble those of the TS4 pathway or align with characteristics of conventional roaming products, which typically exhibit lower moving speeds. Regardless, measuring the speed of internal state-specific H_2_ products appears to be the most effective method for distinguishing the dissociation pathways of the CO_2_ + H_2_ channel.

### 4.6 Limitations of This Study

This section outlines the validity and limitations of the proposed approach. Direct dynamics simulations were conducted without the use of an analytical PES, requiring a trade-off between computational efficiency and the accuracy of the electronic structure methods. Moreover, due to the limited number of trajectories, a quantitative comparison of state-specific data with experimental results could not be performed. Additionally, dissociation pathways were treated as isolated processes, without accounting for connections between multiple pathways. This simplification may not fully reflect realistic conditions. The simulations assume that the molecules always join one of the dissociation pathways before the energy release at the exit barrier, with the distributions of their dynamic variables following the characteristics of the microcanonical ensemble. In other words, the simulations disregarded any nonstatistical behavior of the molecules prior to reaching the saddle point structure. These assumptions may not always hold, especially when molecules arrive in regions other than the minimum of the S_0_ surface through the aforementioned nonradiative decay paths. Regarding the product states of roaming mechanism, there is a difference between the dynamic simulations on analytical PESs and the on-the-fly approach adopted in this study. For dynamic simulations with an analytical PES, the starting structure can be a potential energy minimum of parent molecule, with the initial conditions generated by considering a statistical ensemble at the same structure. Furthermore, such calculation is more realistic, for it not only includes a whole roaming trajectory in simulation, but also avoids assuming a statistical equilibrium of dynamic variables at saddle point. The impact of these two initial conditions on the roaming product states remains unpredictable, but the assumption of statistical equilibrium may be invalid in realistic when molecules return to the S_0_ surface at a structure with one elongated bond.

## 5 Conclusion

This study employed direct dynamics simulations and the Generalized Multi-Center Impulsive Model (GMCIM) to investigate the CO + H_2_O and CO_2_ + H_2_ dissociation channels of formic acid at a photolysis wavelength of 230 nm. The product state distributions of six dissociation pathways, including two roaming pathways, were determined through dynamic calculations performed near the barrier top of each pathway. The characteristics of the product states were analyzed based on the results for each pathway. Comparison with state-specific experimental results at 230 nm suggest that the TS2 and TS5 pathways both play e significant roles in the experimental observation of CO products. At high J(CO) values, the high-speed products are predominantly dissociated via a pathway with a three-center saddle point (TS2). For low-J (CO) states, the OH-roaming pathway (TS5) contributed to the fast component of the experimental speed distribution rather than the slow component. Additionally, the contribution of the isomerization-mediated pathway (TS14) was negligible in the experimental results for both high and low J(CO) states. The relationship between the product state distribution, dissociation pathway, and nonradiative decay paths is discussed. This study clarifies the correlation between dissociation pathways and the characteristics of the product states but does not elucidate the impact of nonradiative decay paths on the dissociation mechanism. Nonadiabatic dynamic simulations are required to gain further insights, regarding the determination of CO_2_ + H_2_ dissociation pathways, our findings indicate that measuring the state-specific speed distribution of H_2_ products in the experiment is necessary.

## Supporting Information Available

Basis function dependence of electronic structure calculation, geometric parameters of saddle point structures, number of successful trajectories in each pathway, starting geometries and IRC profiles of roaming pathways, product state distributions of each pathway, and speed distributions of CO-forming pathways (PDF)
