# Supplementary material for: Photodissociation Dynamics of Formic Acid at 230 nm: A Computational Study of the CO and CO2 Forming Channels
Source: J Phys Chem A. 2025 Jan 16;129(4):1068–84. doi: 10.1021/acs.jpca.4c07933 (PMC11789143; doi:10.1021/acs.jpca.4c07933)
Supplement: Supplementary file 1 — jp4c07933_si_001.pdf [file jp4c07933_si_001.pdf]

Supporting Information

Photodissociation Dynamics of Formic Acid at 230nm: A Computational Study of the CO and CO_2 forming Channels

Yi-Sin Ku and Po-Yu Tsai*

Department of Chemistry, National Chung Hsing University,

Taichung 402, Taiwan

*To whom correspondence should be addressed.

Email: pytsai@nchu.edu.tw

Additional Tables and Figures

TableS1

The energies of the stationary points relative to trans-HCOOH(MIN1) calculated by using M06-2X functional with various basis sets. [kcal/mol]

	**pcseg-1**	**aug-pcseg-1**	**pcseg-2**	**aug-pcseg-2**	***CASPT2**
**MIN2**	5.5	4.4	4.5	4.3	4.2
**MIN4**	41.6	40.5	41.2	41.1	43.4
**MIN5**	50.0	48.3	49.1	48.7	51.2
**$CO+H_2O$**	14.2	12.0	11.8	11.5	7.8
**CO_2+H_2**	2.7	4.0	3.3	3.15	4.2
**TS2**	76.7	74.6	74.8	74.5	70.7
**TS4**	105.3	105.0	106.4	106	98.9
**TS5**	112.9	113.0	114.1	113.9	107.2
**TS9**	76.6	75.8	76.1	75.8	73.7
**TS14**	79.1	78.0	79.2	79.0	77.6
**TS16**	86.1	87.0	88.2	88.3	81.0

* Results of MS-CASPT2/aug-cc-pVDZ were adopted from reference 17 in the main text

TableS2

The geometries of the saddle points TS2 and TS5 calculated by using M06-2X density functional with various basis sets. The atomic distances and angles are reported in Å and degree respectively. The definition of the atomic labels for the geometric parameters is shown in Figure S1.

**TS2**					
	***CASPT2**	**pcseg-1**	**aug-pcseg-1**	**pcseg-2**	**aug-pcseg-2**
C(1)O(2)	1.16	1.14	1.14	1.14	1.14
C(1)H(3)	1.16	1.19	1.18	1.17	1.17
H(3)O(4)	1.43	1.37	1.39	1.39	1.39
O(4)H(5)	0.98	0.97	0.97	0.96	0.96
H(3)C(1)O(2)	168	167	168	168	169
O(4)H(3)C(1)	92	89	90	90	91
H(5)O(4)H(3)	119	126	120	122	121
H(5)O(4)H(3)C(1)	81	91	91	91	91
**TS5**					
C(1)O(2)	1.2	1.18	1.18	1.17	1.17
C(1)H(3)	1.12	1.13	1.12	1.12	1.12
O(2)O(4)	2.9	2.72	2.72	2.73	2.75
O(4)H(5)	0.98	0.98	0.98	0.97	0.97
H(3)C(1)O(2)	124	124	124	125	125
O(4)O(2)C(1)	83	78	79	80	79
H(5)O(4)O(2)	61	67	66	70	68
O(4)O(2)C(1)H(3)	-69	-71	-66	-70	-67
H(5)O(4)O(2)C(1)	162	168	164	176	177

* Results of MS-CASPT2/aug-cc-pVDZ were adopted from reference 17 in the main text

TableS3

The geometries of the saddle points TS4, TS9, TS14 and TS16 calculated at the M06-2X/aug-pcseg-1 level. The atomic distances and angles are reported in Å and degree respectively. The definition of the atomic labels for the geometric parameters is shown in Figure S1.

TS9			TS14		
	*CASPT2	M06-2X		*CASPT2	M06-2X
C(1)O(2)	1.19	1.17	C(1)O(2)	1.67	1.61
C(1)H(3)	1.43	1.44	O(2)H(3)	0.97	0.97
C(1)O(4)	1.28	1.25	C(1)O(4)	1.26	1.23
H(3)H(5)	1.03	1.03	O(4)H(5)	1.20	1.22
H(3)C(1)O(2)	113	113	H(3)O(2)C(1)	112	114
O(4)C(1)H(3)	101	100	O(4)C(1)O(2)	92	93
H(5)H(3)O(4)	33	32	H(5)O(4)O(2)	34	34
H(5)H(3)O(4)C(1)	180	180	O(4)C(1)O(2)H(3)	-122	-124
			H(5)O(4)O(2)C(1)	-171	-172
**TS16**			**TS4**		
	*CASPT2	M06-2X		*CASPT2	M06-2X
C(1)O(2)	1.24	1.22	C(1)O(2)	1.20	1.18
C(1)H(3)	1.90	1.89	C(1)H(3)	2.78	2.71
C(1)O(4)	1.24	1.22	C(1)O(4)	1.34	1.32
H(3)H(5)	1.11	1.07	O(4)H(5)	0.98	0.98
H(3)C(1)O(2)	78	78	H(3)C(1)O(2)	80	84
O(4)C(1)H(3)	44	45	O(4)C(1)O(2)	130	130
H(5)H(3)O(2)	36	38	H(5)O(4)C(1)	108	109
H(5)H(3)O(2)C(1)	180	180	O(4)C(1)O(2)H(3)	-73	-72

* Results of MS-CASPT2/aug-cc-pVDZ were adopted from reference 17 in the main text

Table S4 Number of trajectories of direct dynamic simulations performed in this study. Values with parenthesis denotes the total number of trajectories, while values without parenthesis are the number of the trajectories which form the products successfully. Values in the square bracket are the numbers of remaining trajectories after excluding the trajectories that with Ev(CO)<ZPE(CO).

	**ZPE**	**248nm**	**230nm**
**TS2**	999 (1000)	1980 (2000)	5944 (6000) [4142]
**TS14**	997 (1000)	-	2980(3000)[2212]
**TS5**	1999 (2102)	-	5961(8799)[3970]
**TS9**	999 (1000)	992 (1000)	1983(2000)
**TS16**	1986 (2000)	-	1972(2000)
**TS4**	984 (1000)	-	2512(3170)
**sum**	7964(8102)	2972 (3000)	21352(24969)

Table S5 mean values and standard deviations of the dissociation times in direct dynamic simulations

	**ZPE**	**248nm**	**230nm**
**TS2**	90±18	77±19	76±20
**TS14**	78±6	-	78±11
**TS5**	166±38	-	177±34
**TS9**	39±4	36±7	36±6
**TS16**	46±4	-	51±9
**TS4**	57±13	-	67±24

TableS6 Energies in kcal/mol, of the stationary points relative to trans-HCOOH(MIN1), either obtained from experiments or calculated at various computational levels. The values with parenthesis are the ones with zero point energy being included.

	**TS9**	**TS2**	**CO_2+H_2**	**CO+H_2O**
**MP2/cc-pvdz**	71.7	72.9	-4.6	10.2
	(65.5)	(67.9)	(-13.3)	(5.4)
**M06-2X/aug-pcseg-1**	75.8	74.6	4.0	12.0
	(69.8)	(70.0)	(-4.5)	(7.3)
**MP2/cc-pvdz[1]**	(65.6)	(67.9)	(-12.4)	(5.4)
**CASPT2/aug-cc-pvdz[2]**	73.7	70.7	4.2	7.8
**B3LYP/6-311++G(3df,3dp)[1]**	(66.6)	(64.6)	(-6.7)	(8.1)
**Experiment[1]**	-	-	(-3.6)	(6.3)
**Experiment[1]**	(60.6)	-	(-5.2)	-

[1] Results were adopted from Table 2 of Reference 57 (see also the references therein) of the main text

[2] Results were adopted from Reference 17 of the main text

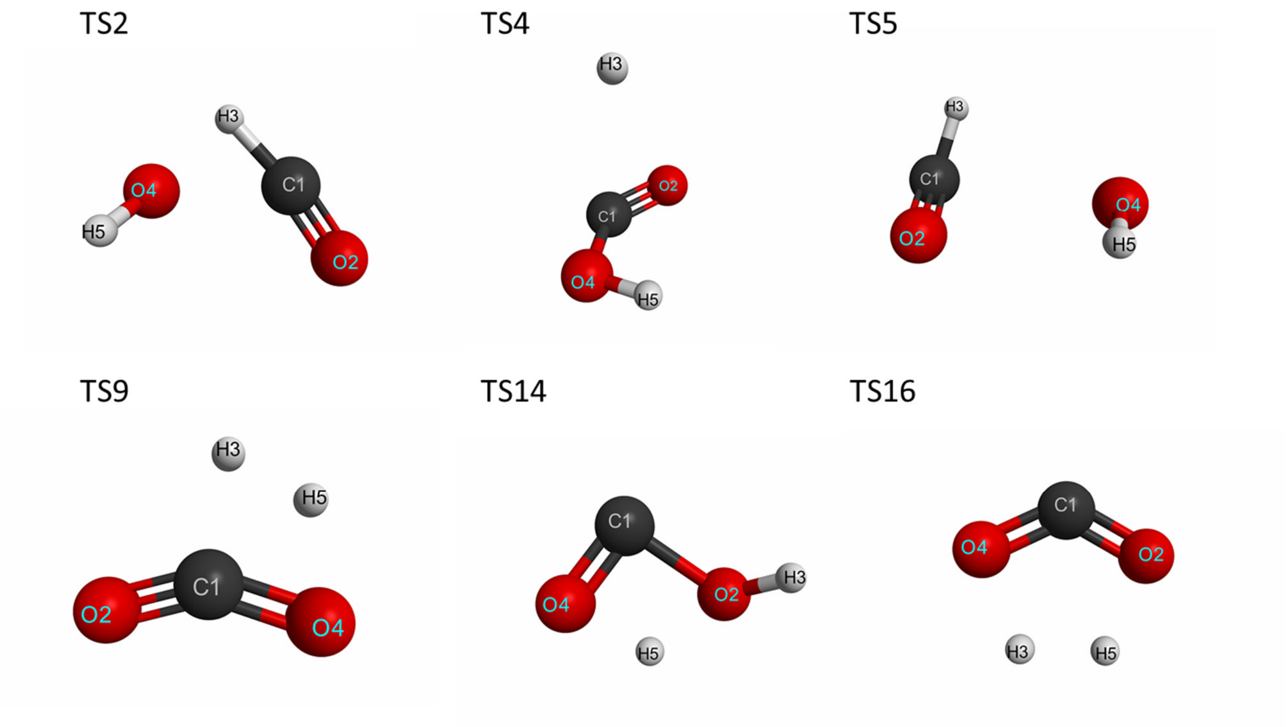

Figure S1 atomic labels of each saddle point structure defined for the geometric parameters in Table S2 and Table S3.

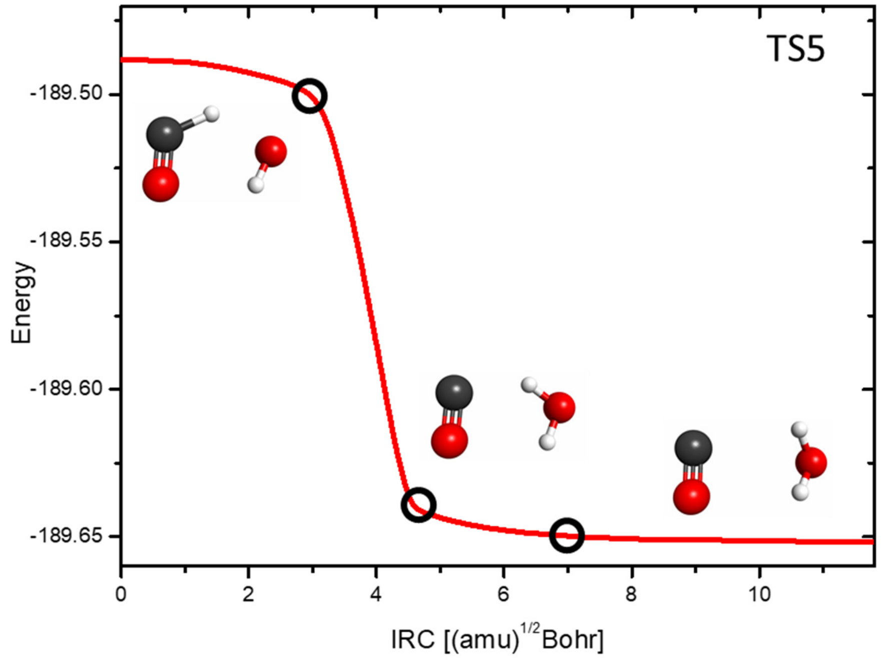

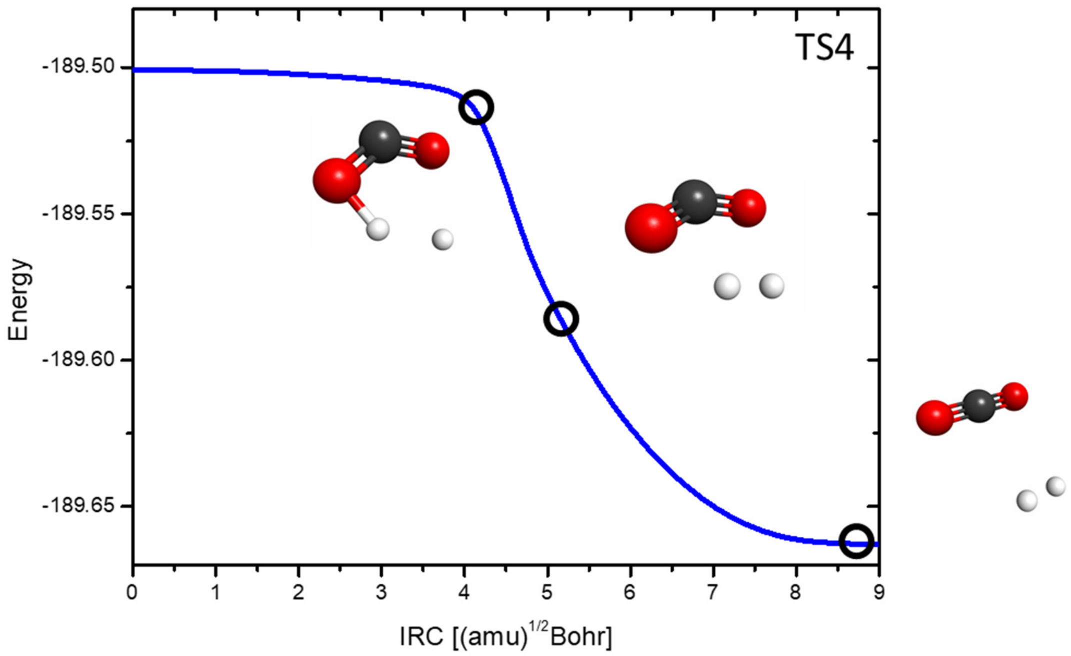

Figure S2 The IRC profiles of TS5 and TS4 pathways, from saddle points (at 0) toward the products, calculated at the uM06-2X/aug-pcseg-1 level. The three circles mark the regions of IRC where the molecular structures are also shown in the each plot. The rapid drop of potential energy of TS5 pathway is due to the decrease of H--OH distance without varying the center of mass (c.m.) positions of CO and H_2O moieties. For TS4 pathway, however, the rapid drop of potential energy can be attributed to both the

decreasing of H—H distance (first half) and the increasing of c.m. positions of the two moieties(second half).

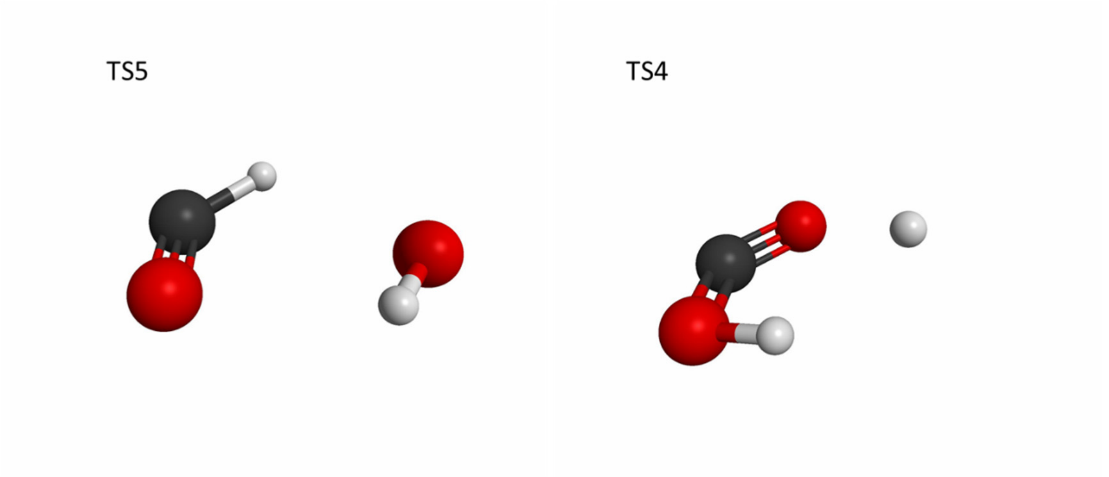

Figure S3 The starting structures of the two roaming pathways adopted in the simulations of direct dynamics and GMCIM. These two structures correspond to the configurations of the IRC at 1.97 and 3.50 $(amu)^{1/2}$-Bohr downhill from the saddle points TS5 and TS4, respectively; both structures were obtained at level of uM06-2X/aug-pcseg-1.

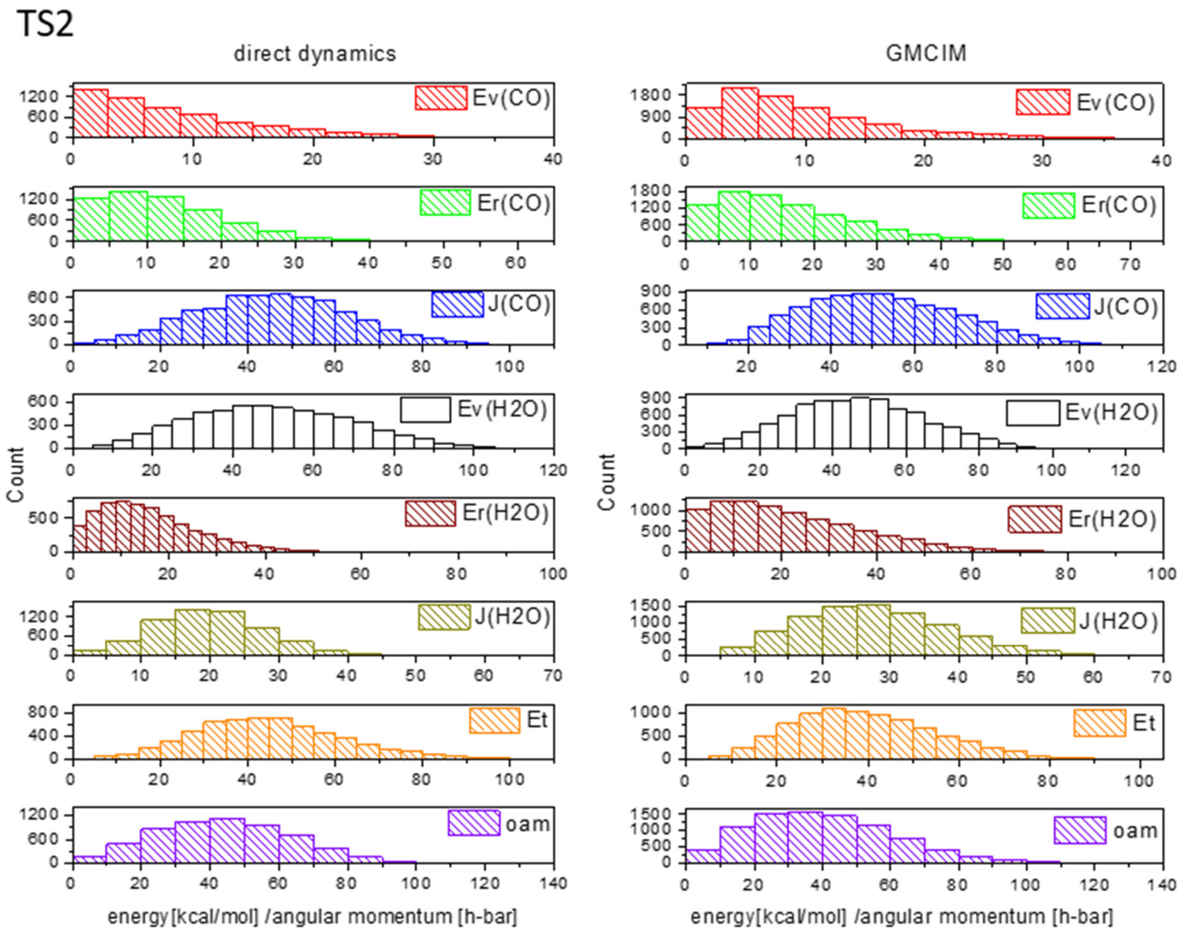

Figure S4 The product state distributions from the direct dynamics simulation and GMCIM of TS2 pathway: translational energy (E_T), vibrational energy (E_V), rotational angular momentum (J), and angular momentum of fragments orbiting motion (OAM) are shown in the figure.

TS14

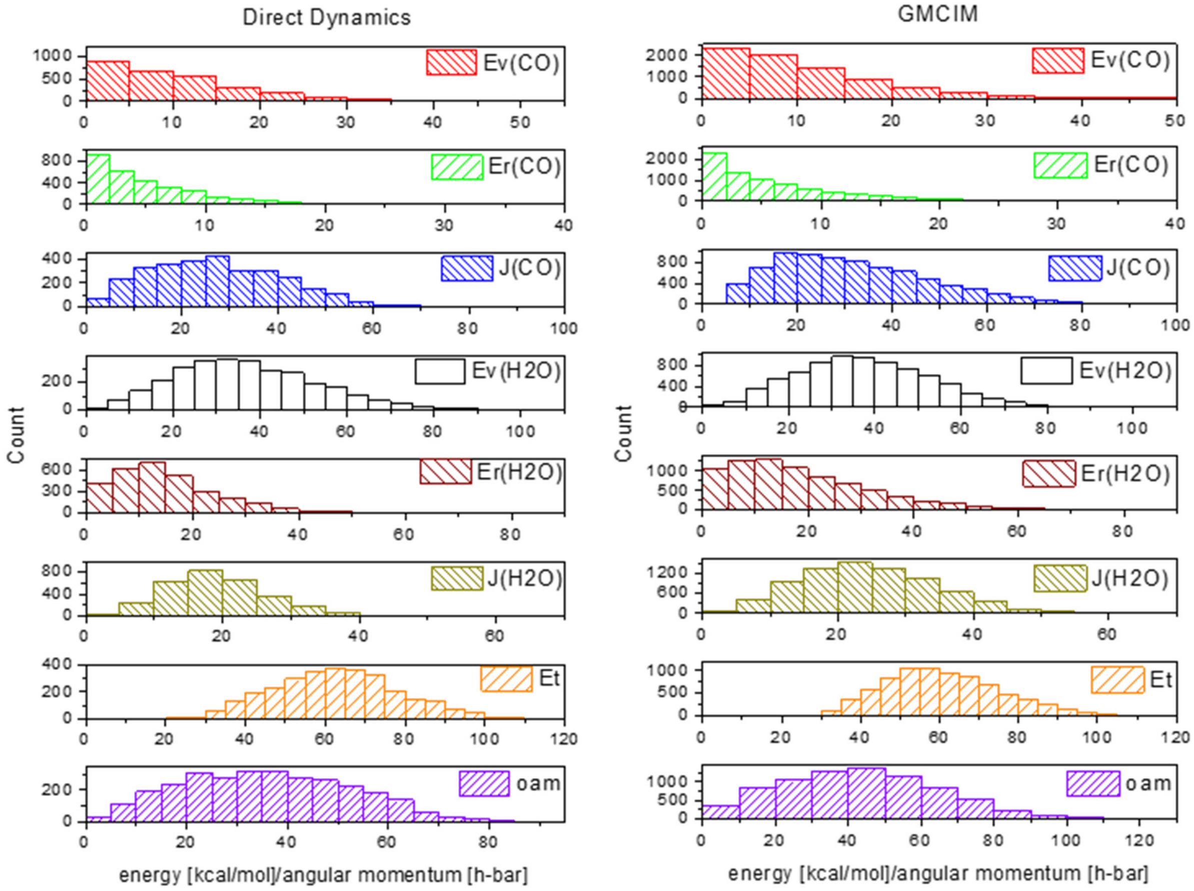

Figure S5 The product state distributions from the direct dynamics simulation and GMCIM of TS14 pathway: translational energy (ET), vibrational energy (EV), rotational angular momentum (J), and angular momentum of fragments orbiting motion (OAM) are shown in the figure.

TS5

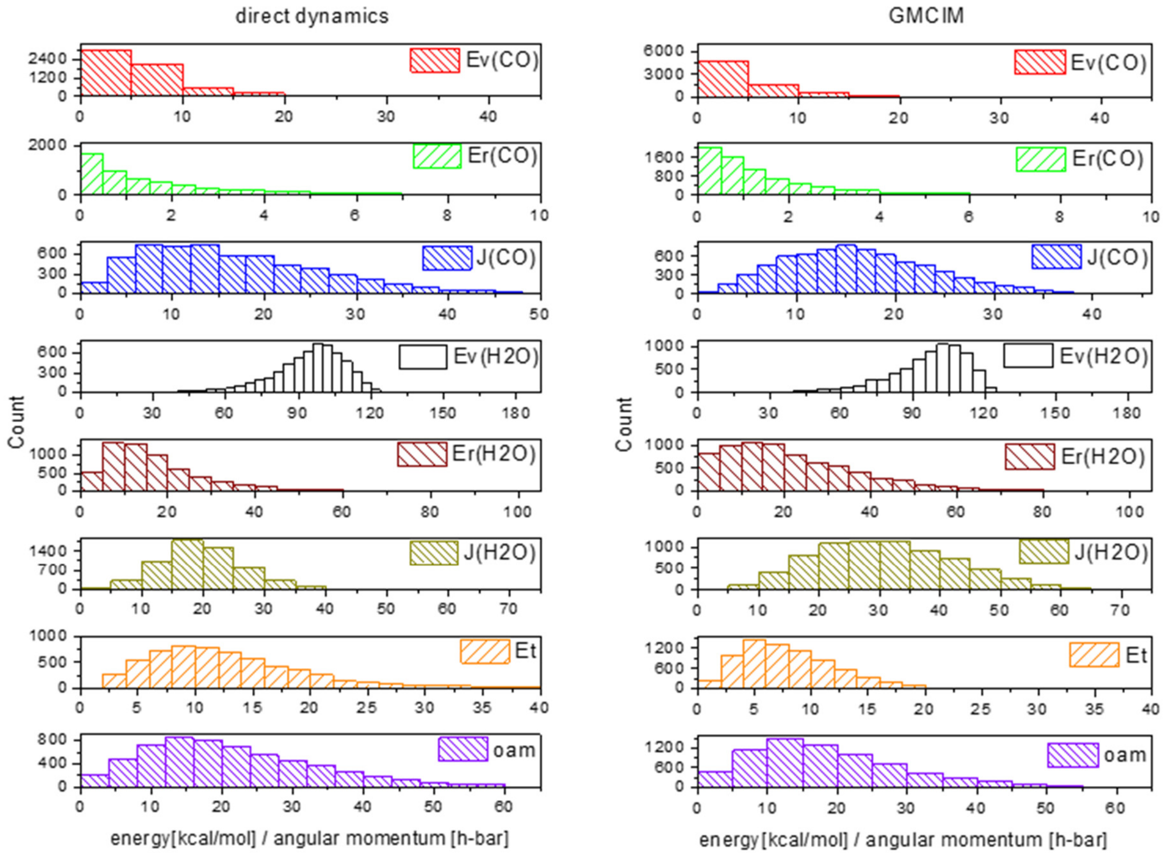

Figure S6 The product state distributions from the direct dynamics simulation and GMCIM of TS5 pathway: translational energy (ET), vibrational energy (EV), rotational angular momentum (J), and angular momentum of fragments orbiting motion (OAM) are shown in the figure.

TS9

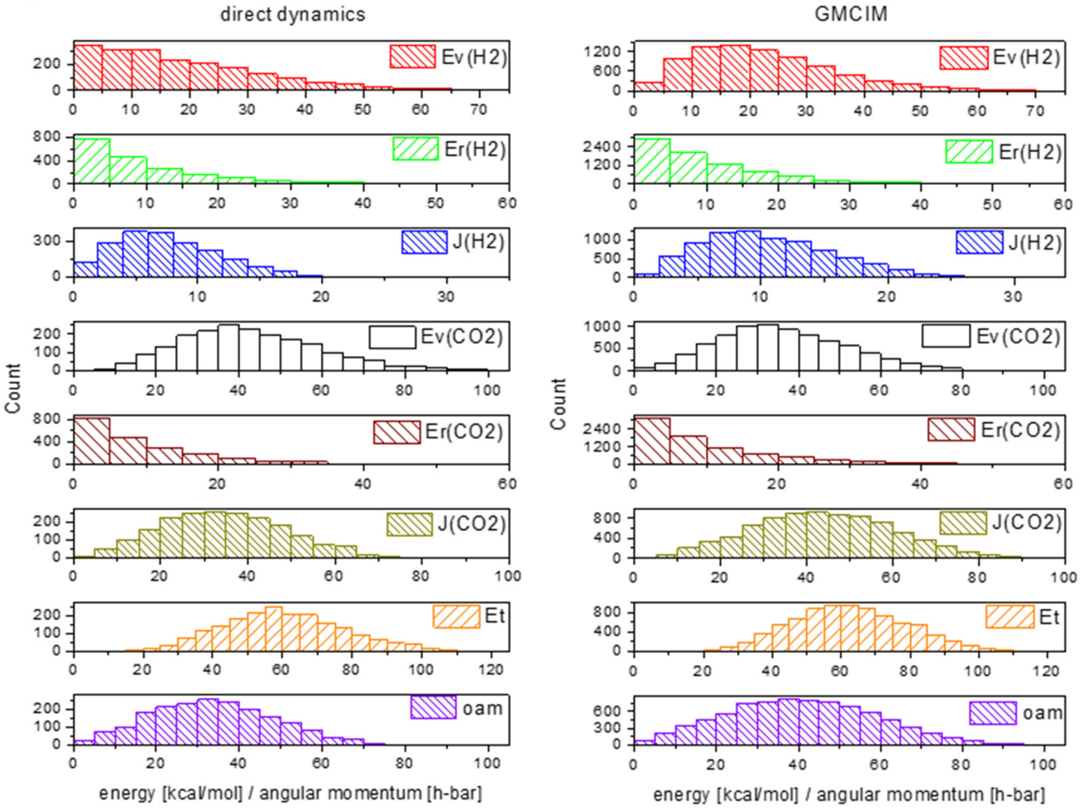

Figure S7 The product state distributions from the direct dynamics simulation and GMCIM of TS9 pathway: translational energy (E_T), vibrational energy (E_V), rotational angular momentum (J), and angular momentum of fragments orbiting motion (OAM) are shown in the figure.

TS16

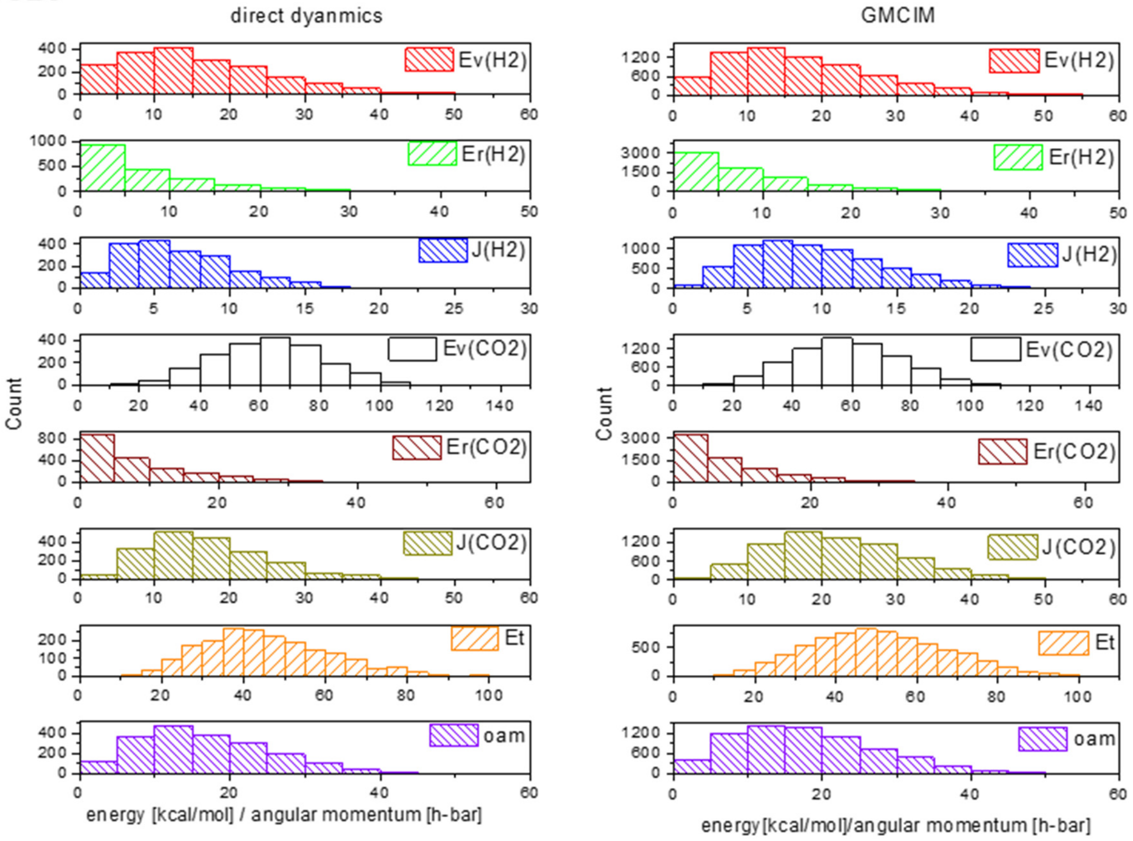

Figure S8 The product state distributions from the direct dynamics simulation and GMCIM of TS16 pathway: translational energy (E_T), vibrational energy (E_V), rotational angular momentum (J), and angular momentum of fragments orbiting motion (OAM) are shown in the figure.

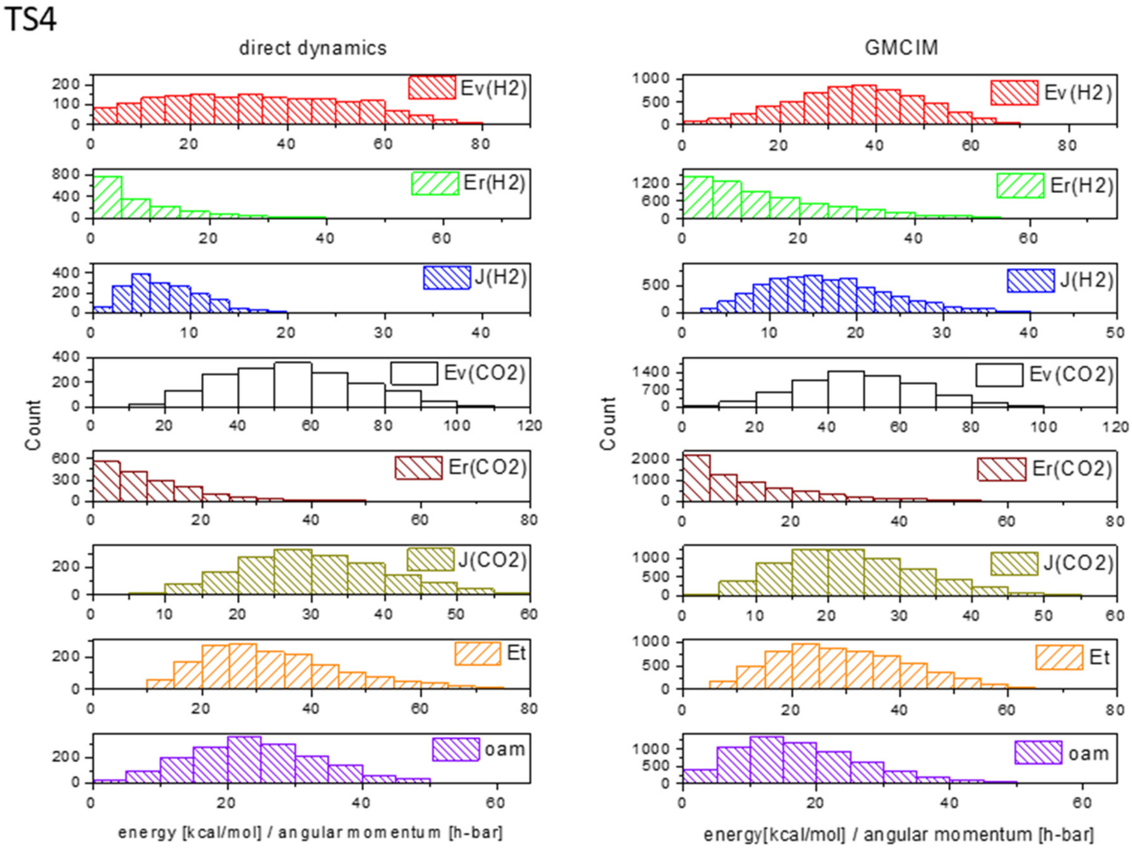

Figure S9 The product state distributions from the direct dynamics simulation and GMCIM of TS4 pathway: translational energy (E_T), vibrational energy (E_V), rotational angular momentum (J), and angular momentum of fragments orbiting motion (OAM) are shown in the figure.

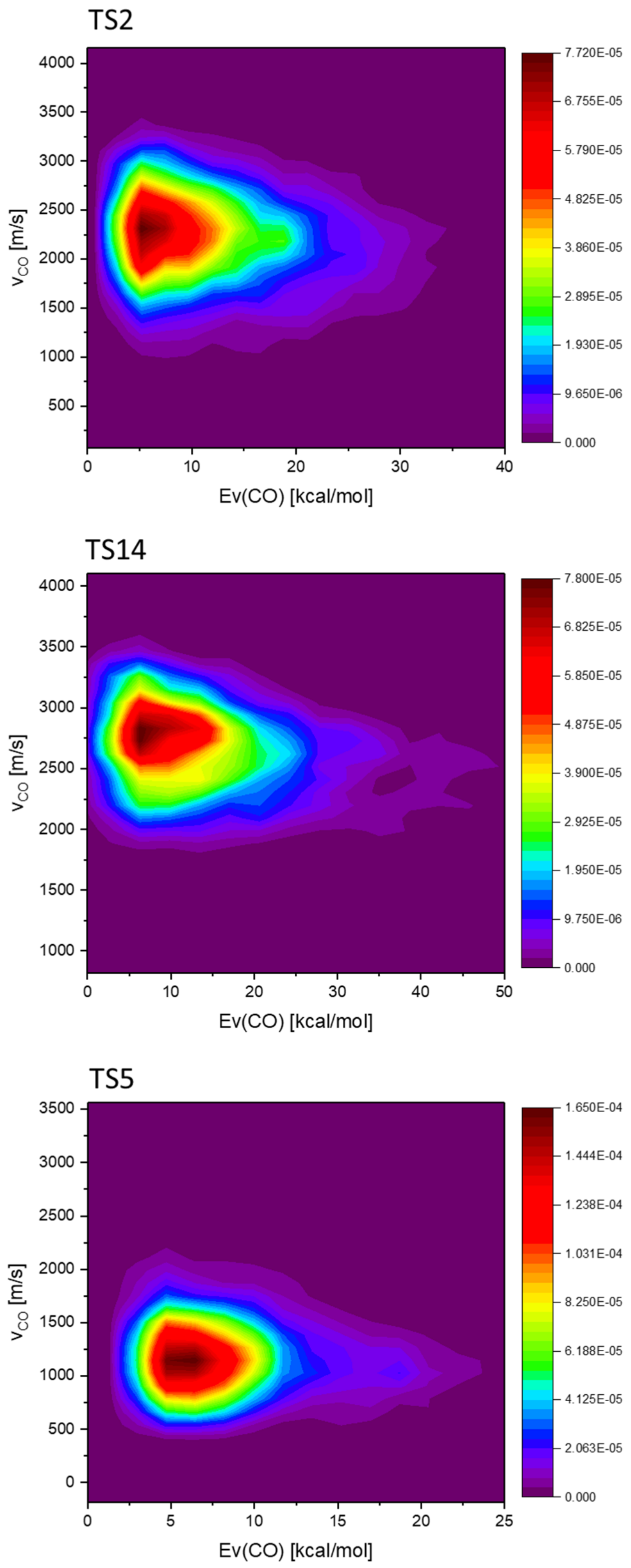

Figure S10 v_{CO} vs. Ev(CO) correlation diagrams of TS2, TS14 and TS5 pathways. The most probable velocity of CO (v_{CO}) is almost unchanged with respect to vibrational energy of CO (Ev(CO)) in each pathway. The trajectory events which violate zero point level of CO products have been eliminated before making these plots.

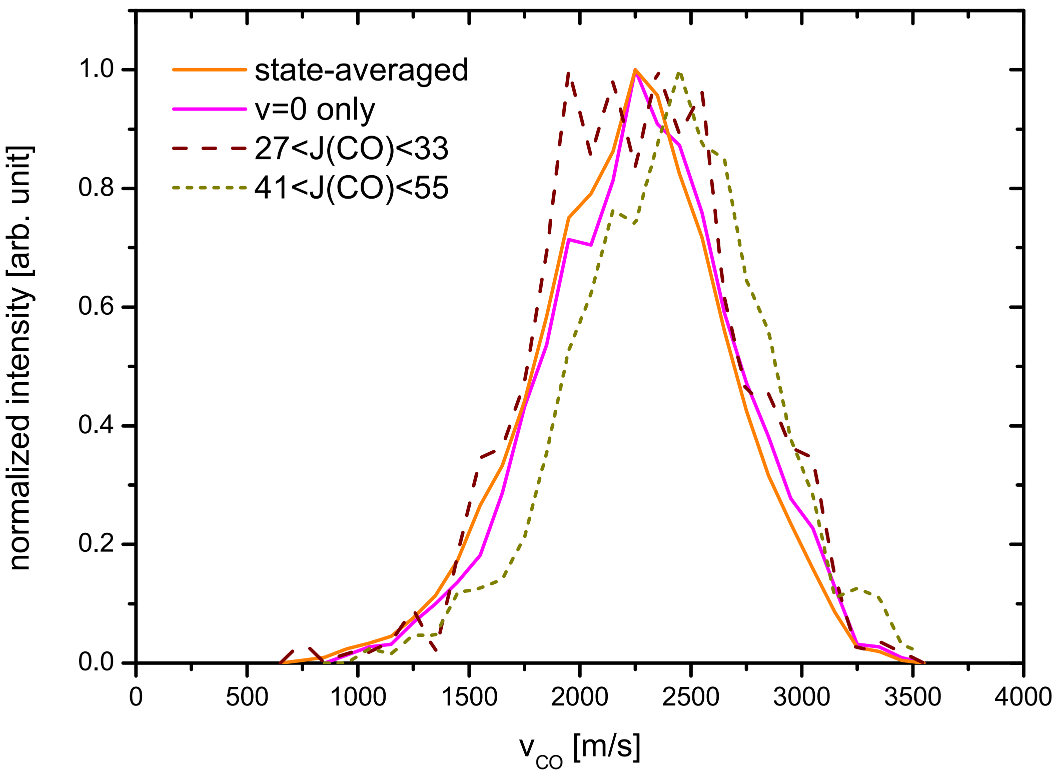

Figure S11 The speed distributions of CO from TS2 pathway, with or without the vibrational or rotational state-selections to CO products. The binning size of histograms is 100 m/s.
